# PRognostic and predictive potential Of multiparametric dynamic whole-body 18F-FDG PET Imaging using a Long axial field-of-view (LAFOV) system for FIRST-line chemo-immunotherapy efficacy in advanced non-small cell lung cancer: PROFIL-1 study protocol

**DOI:** 10.1371/journal.pone.0345990

**Published:** 2026-04-03

**Authors:** Margaux Geier, Karim Amrane, David Bourhis, Pierre-Yves Le Roux, Jessica Nguyen, Estelle Dhamelincourt, Renaud Descourt, Gilles Quéré, Marie Guegan, Benjamin Besse, Désirée Déandreis, Florent Besson, Sylvain Faure, Nicolas Karakatsanis, Pierre-Yves Salaun, Mathieu Pavoine, Vincent Bourbonne, François Lucia, Ronan Abgral

**Affiliations:** 1 Univ Brest, Inserm, UMR 1304, GETBO, Brest, France; 2 Department of Medical Oncology, Cancer & Imaging Institute, CHU La Cavale Blanche, University Hospital of Brest, Brest, France; 3 Department of Medical Oncology, CH Morlaix, Morlaix, France; 4 Department of Molecular Imaging and Theranostics, Cancer & Imaging Institute, CHU La Cavale Blanche, University Hospital of Brest, Brest, France; 5 Department of Medical Oncology, International Center for Thoracic Cancers (CICT), Gustave Roussy, Villejuif, France; 6 Paris-Saclay University, Paris, France; 7 Imaging Department, Nuclear Medicine Service, Gustave Roussy, Villejuif, France; 8 Department of Nuclear Medicine and Molecular Imaging, Paris-Saclay University, AP-HP, CHU Bicêtre, DMU SMART IMAGING, Le Kremlin-Bicêtre, Paris, France; 9 Laboratoire de Mathématique d’Orsay, CNRS, Université Paris-Saclay, Orsay City, France; 10 Department of Radiology, Weill Cornell Medical College, New York, New York, United States of America; 11 Radiation Oncology Department, University Hospital, Brest, France; 12 LaTIM, INSERM, UMR 1101, University of Brest, ISBAM, UBO, UBL, Brest, France; Fondazione Policlinico Universitario Agostino Gemelli IRCCS, ITALY

## Abstract

**Background:**

Revolution of chemo-immunotherapy (CT-IO) in the first-line treatment of metastatic non-small-cell lung cancers (NSCLC) without actionable genomic alterations (AGAs) has dramatically improved prognosis, providing long response in a subset of patients. Due to the highly heterogeneous nature of the disease, most of patients do not show long term benefit. Long axial field of view positron emission tomography (LAFOV-PET) scanner is a new emerging system allowing dynamic whole-body imaging with higher sensitivity, representing unique opportunity for oncological applications. The aim of this study is to determine whether ^18^F-fluorodeoxyglucose positron emission (^18^F-FDG) LAFOV-PET derived parameters might have prognostic and predictive potential for CT-IO outcomes in NSCLC.

**Methods:**

PROFIL-1 (NCT06738680) is a multicentre, prospective single-arm biomarker pilot study investigating the prognostic and predictive potential of multiparametric ^18^F-FDG LAFOV-PET for first-line CT-IO efficacy in advanced NSCLC, with a planned enrolment of 120 patients at 2 French sites. Adult patients with treatment-naïve advanced non-squamous or squamous NSCLC without AGAs and eligible for first-line CT-IO will be recruited for PROFIL-1. Patients will undergo baseline LAFOV-PET before treatment (optional second dynamic LAFOV-PET after CT-IO induction). The primary objective is to evaluate the prognostic and predictive potential of a whole-body multiparametric analysis (radiomics and dynamics) derived from LAFOV-PET for first-line CT-IO efficacy, using the rate of disease progression or death at one year as the primary endpoint, assessed by investigators according to RECIST v1.1 criteria. Secondary endpoints included correlations between imaging parameters and clinico-pathological characteristics, comparison between direct Patlak and indirect Patlak reconstruction methods to determine dynamic parameters such as Ki (the net influx rate) and distribution volume (DV), number of detected tumor lesions and signal-to-noise ratio (vs. SAFOV-like imaging), objective response rate, overall survival and safety.

The study opened for enrolment in January 2025. Duration of inclusions: 2 years.

**Clinical trial information:**

NCT06738680.

## Introduction

### Context

Lung cancer is the most diagnosed cancer and the leading cause of cancer deaths worldwide, in both men and women [1]. Because lung cancer is often detected at an advanced stage beyond curative treatment, the five-year survival rate remains poor, less than 20% in most countries [2]. In the last decade, new treatment modalities including targeted therapies and immune checkpoint inhibitors (ICI), have provided the opportunity to improve outcomes for patients with non-small cell lung cancer (NSCLC). First-line chemo-immunotherapy (CT-IO) has notably demonstrated remarkable response rates and survival benefits in metastatic NSCLC without actionable genomic alterations (AGAs) [3,4]. Nonetheless, due to the highly heterogeneous nature of the disease, most of patients treated with ICI do not show long term benefit. Programmed death-ligand 1 (PD-L1) expression on tumour cells remains the only predictive biomarker in daily practice, but does not fully explain the sensitivity and resistance mechanisms to ICI [5].

### Current knowledge

Different types of solid tumours, including NSCLC, exhibit high inter- and intra-tumoral heterogeneity (cell clones with different proliferation rates and different areas of angiogenesis, hypoxia, necrosis, or fibrosis, etc.), which may explain differences in systemic treatment efficacy and predispose patients to worse clinical outcomes [6]. Assessing tumour heterogeneity is a major challenge in oncology to improve therapeutic management and move towards personalized medicine tailored to each patient.

^18^F-fluorodeoxyglucose positron emission tomography-computed tomography (^18^F-FDG PET/CT) is a functional imaging technique that measures tumour glucose metabolism and is currently widely used in the management of lung cancer, especially for response assessment after systemic treatment [**7**]. The search for surrogate markers in PET/CT imaging for early prediction of response to treatment and survival is progressing [8–10]. Indeed, the means of ^18^F-FDG PET/CT semiquantitative measures, such as tumour burden (metabolic tumour volume (MTV), total lesion glycolysis (TLG)), are emerging as future evidence in monitoring immunotherapy efficacy. Recently, long axial field of view positron emission tomography (LAFOV-PET) scanner has emerged as an innovative next-generation system allowing dynamic whole-body imaging with higher sensitivity, leading to an improvement in the detectability of lesions and thus representing a unique opportunity for oncological applications [11]. These features highlight real possibilities for optimizing the multi-parametric analysis of PET images.

4D dynamic analysis (dynPET) has been proposed to extract quantitative parameters from the temporal analysis of the radiotracer distribution in voxels [12,13]. It allows the estimation of kinetic parameters by modelling based on a Patlak-like analysis after estimating the plasma input function from images (Image Based Input Function, IDIF) or a control population (Population Based Input Function, PBIF) [14]. Initial studies on dynPET acquisitions have shown the absence of a linear correlation between SUV and Ki values [15], suggesting the contribution of additional quantitative information from kinetic data and opening new perspectives for the prognostic assessment of NSCLC [16].

Texture analysis (radiomics) in PET/CT imaging, which corresponds to an analysis of the spatial distribution of voxels, allows the calculation of numerous indices reflecting tumour heterogeneity [17]. Some studies have demonstrated the prognostic value of texture analysis in ^18^F-FDG PET/CT for NSCLC [18]. However, the quality of this texture analysis depends on image noise (and therefore on the intrinsic performance of the system) and remains limited for small or mobile lesions, such as in the lung [19].

LAFOV-PET could overcome these current limitations with the possibility of image reconstruction using a high-resolution matrix with limited noise and excellent temporal sampling (whole-body in 10–20 seconds) to optimize radiomic analysis and 4D dynamic quantification. Especially, in lung cancer, only one recent study has shown the potential interest of this multiparametric analysis technique in LAFOV-PET to predict response to induction CT-IO. However, the analysis was performed only on the primary tumours in patients with locally advanced NSCLC [20].

### Study and aims

Non-invasive predictors of first-line CT-IO outcome in metastatic NSCLC are needed. Beyond the importance of cancer health economics, the early identification of refractory patients would be of undeniable benefit to their survival, leading to the identification of a “high-risk subgroup” requiring therapeutic escalation. To address this issue, we hypothesize that LAFOV-PET derived parameters could improve the prediction of CT-IO efficacy in advanced NSCLC patients.

## Hypotheses and objectives

### Study hypotheses

We hypothesise that LAFOV FDG-PET may improve early prediction of outcomes to first-line CT-IO in patients with advanced NSCLC in addition to conventional biomarkers.

### Objectives

The primary objective is to evaluate the prognostic performance and predictive potential of a whole-body multiparametric analysis (radiomics and dynamics) in LAFOV-PET on CT-IO efficacy based on investigator assessment of progression-free survival (PFS) according to RECIST criteria [21].

Secondary objectives:

1. To investigate the association between LAFOV-PET quantitative dynamic and radiomic parameters and clinico-histopathological parameters (clinical, biological, histological, genomic, etc.).
2. To compare direct and indirect Patlak methods with both image-derived input function (IDIF) and population-based input function (PBIF) in LAFOV-PET for parameters calculation.
3. To compare quantitative parameters resulting from LAFOV-PET acquisition with those resulting from a degraded post-acquisition reconstruction short axial field-of-view (SAFOV) PET “like”.
4. Therapeutic response.
5. Overall survival (OS).
6. Safety.

## Methods: Participants, interventions and outcomes

### Trial design

PROFIL-1 (NCT06738680) is a multicentre, prospective single-arm biomarker pilot study. This manuscript describes protocol version 1.0 of the PROFIL-1 study, dated March 14, 2024, and approved by the Comité de Protection des Personnes Sud-Est VI on 15 July 2024.

### Study setting

Patients will be recruited from the Cancer and Imaging Institute of the University Hospital of Brest and from the Department of Medical Oncology of the Morlaix Regional Hospital. The study will open for enrolment on January 31, 2025, and close on January 31, 2027. We estimate that recruitment will take around 24 months. Patient’s participation will last 12 months.

### Calculation of the number of subjects required

It would be necessary to establish relevant thresholds for each biomarker (using an ROC curve) and calculate the sensitivity/specificity values obtained for each parameter, with respect to the primary binary endpoint (tumour progression or death at one year). With 120 patients, about 60% of whom will show tumour progression or death at one year, it would be possible to estimate sensitivity/specificity values with an accuracy (1/2 amplitude of the 95% confidence interval) of about 10% for sensitivity and 12% for specificity, assuming observed values of about 80%. This progression rate assumption is supported by published pivotal trial and real-world data in advanced NSCLC treated with first-line chemo-immunotherapy (e.g., KEYNOTE-189 [3] and KEYNOTE-407 [4]). Given the exploratory nature of this study, the sample size is intended to support precision-based estimation of diagnostic/prognostic performance metrics rather than confirmatory hypothesis testing.

### Inclusion and exclusion criteria

The inclusion and exclusion criteria are summarized in **Table 1**.

**Table 1:** Inclusion and exclusion criteria.

Inclusion criteria	Exclusion criteria
• Patients aged ≥ 18 years • ECOG Performance Status 0–2 • Locally advanced unresectable, not eligible for curative-intent radiotherapy, or metastatic NSCLC (based on the American Joint Committee on Cancer (AJCC) staging system (8^th^ edition) • Treatment-naïve patients • Eligible for first-line chemo-immunotherapy (anti-PD-1) • LAFOV-PET < 21 days prior to treatment • Written informed consent provided	• Minor patients• Actionable genomic alterations: *EGFR*, *ALK*, *ROS1*, *RET* • Other/mixed histology • Previous exposure to any anti-PD-1 or anti-PD-L1 antibody • Not eligible for first-line chemo-immunotherapy • PET contraindications or inability to tolerate prolonged supine positioning during the dynamic acquisition • Pregnancy/breastfeeding • Any condition that, in the opinion of the investigator, would interfere with the evaluation of the study drug or the interpretation of patient safety or study results • Refusal to participate

### Who will take the informed consent?

Patients who meet all the inclusion criteria and none of the exclusion criteria will be offered the opportunity to participate in the study and will be given all the relevant information verbally and in writing. Informed consent for the study will be provided by the clinical investigators at each participating site. Patient data were accessed for research purposes starting on January 31, 2025, which corresponds to the initiation of patient inclusion in the PROFIL-1 study. The authors had access exclusively to pseudonymized data, with no direct identifiers of individual participants available during or after data collection.

### Interventions

**Fig 1:**
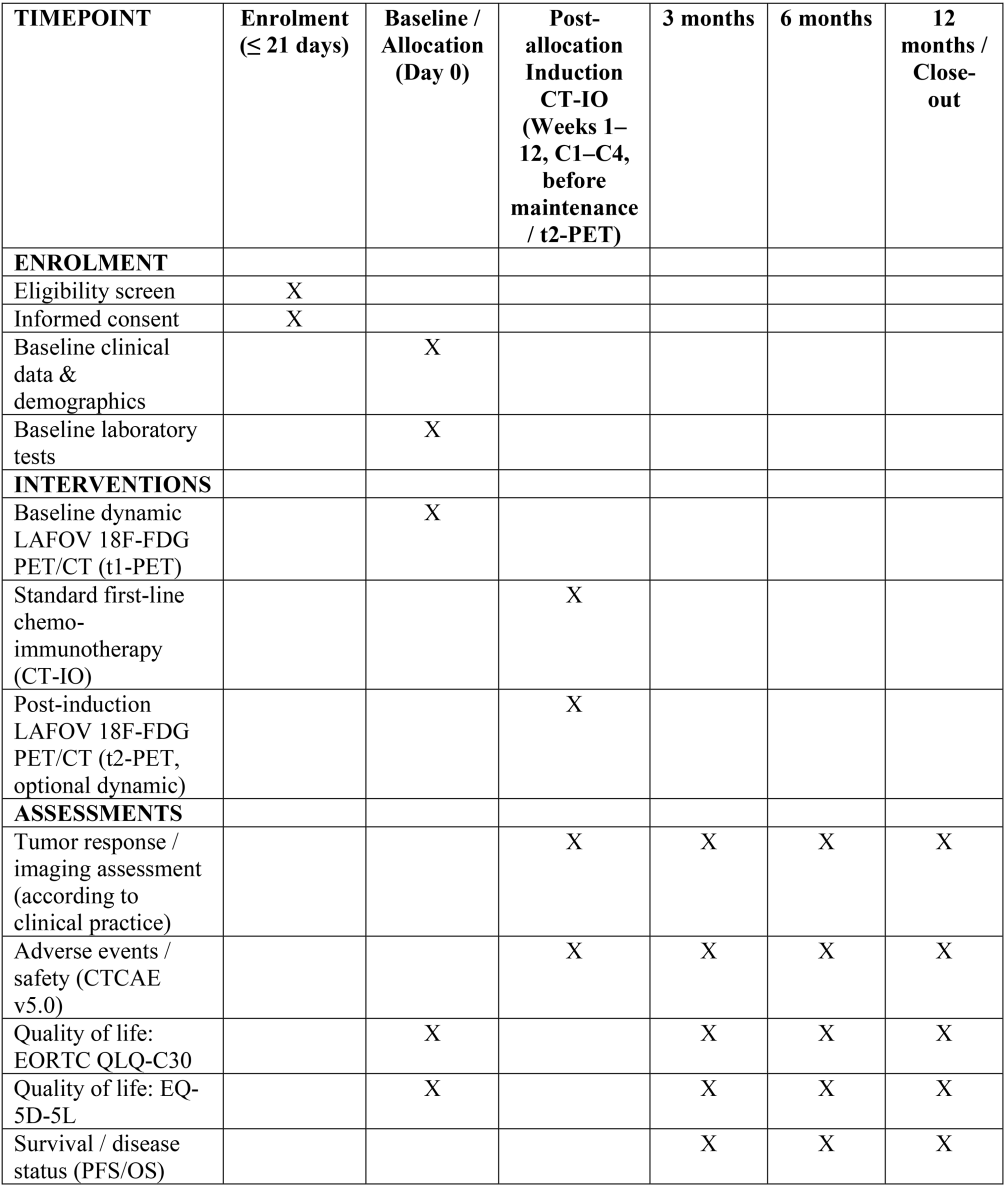
PROFIL-1—SPIRIT Schedule of enrolment, interventions, and assessments. The schedule summarizes participant enrolment, baseline assessments, study interventions, imaging procedures, and follow-up visits at 3, 6, and 12 months. QoL questionnaires (EORTC QLQ-C30 and EQ-5D-5L) are administered at baseline and follow-up time points. An optional post-induction LAFOV PET/CT (t2) may be performed after the fourth cycle of chemo-immunotherapy.

**Fig 2:**
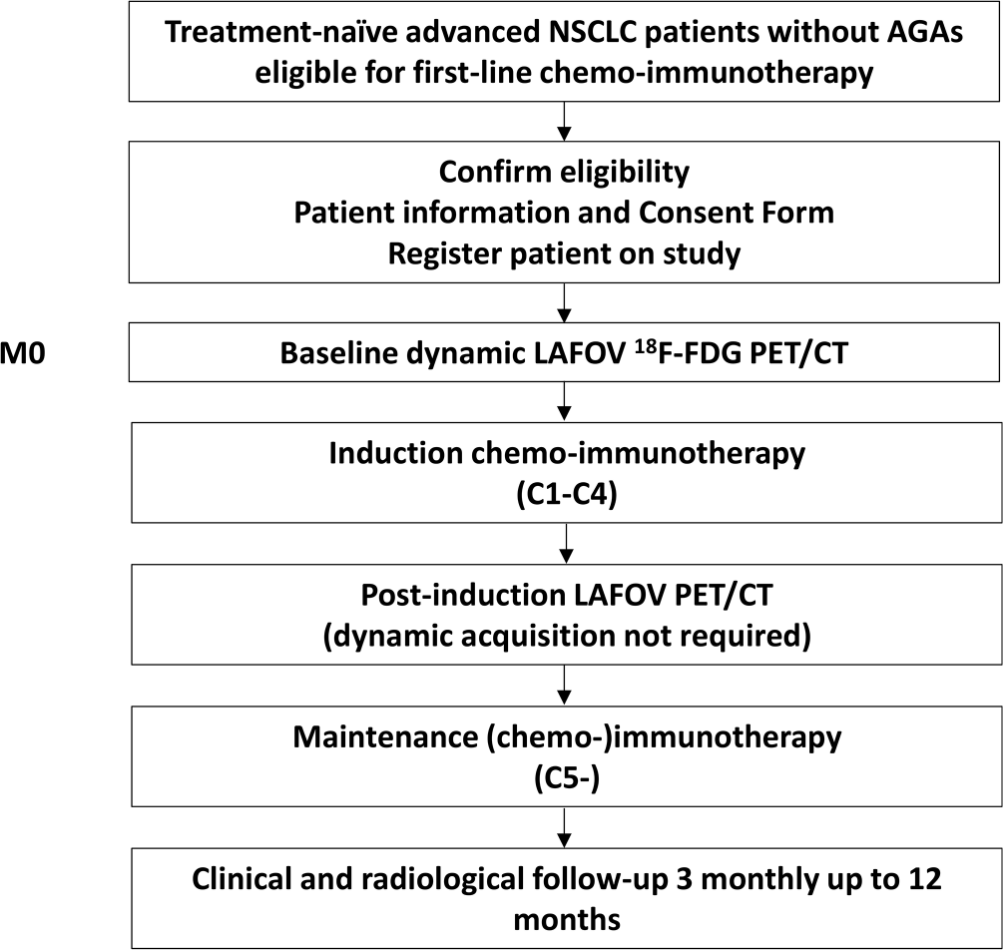
Study flow chart. NSCLC: Non-small cell lung cancer: AGAs: Actionable genomic alterations; LAFOV ^18^F-FDG PET/CT: Long axial field of view ^18^F-fluorodeoxyglucose positron emission tomography-computed tomography.

The schedule of participant enrolment, interventions and assessments is presented in **Figs 1** and **2**. Potential participants will be included at their first visit to an oncologist, prior to baseline dynamic LAFOV PET, after written consent.

#### Chemo-immunotherapy.

Treatment will be administered according to current guidelines. For patients with non-squamous histology, the standard of care is based on a maximum of four cycles of pemetrexed (500 mg/m^2^)-cisplatin (75 mg/m^2^)/carboplatin (AUC5) plus pembrolizumab (200 mg) every three weeks as induction, then pemetrexed-pembrolizumab maintenance every three weeks for two years or until disease progression (DP) or unacceptable toxicity. For patients with squamous histology, the standard of care is based on a maximum of four cycles of paclitaxel (200 mg/m^2^)-carboplatin (AUC6) plus pembrolizumab (200 mg) as induction, then pembrolizumab maintenance for two years or until DP or unacceptable toxicity.

Management of immune-related adverse events (irAEs), including treatment delays, dose modifications or discontinuation, will follow institutional practice and international guidelines (e.g., ESMO). All irAEs and treatment modifications will be prospectively recorded in the electronic case report form.

#### LAFOV PET/CT scan acquisition and analysis.

**Fig 3:**
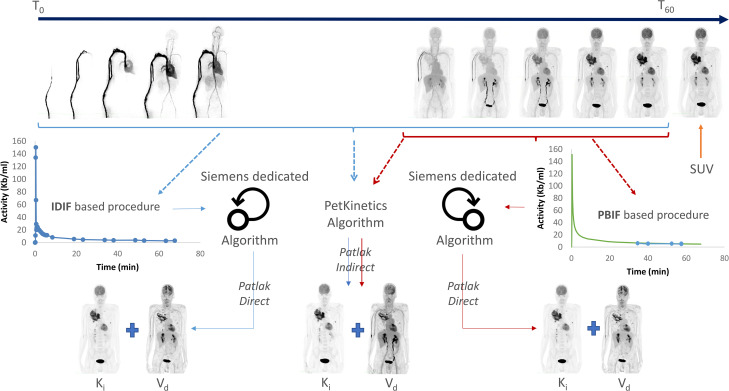
Example for an acquisition protocol for a dynamic acquisition over one bed position followed by Patlak reconstructions with IDIF and PBIF based procedures with the Biograph Vision Quadra^TM^.

Baseline whole-body dynamic (WBdyn) LAFOV ^18^F-FDG PET/CT scan (t1-PET) will be performed within 21 days prior to the first cycle of chemo-immunotherapy induction, as part of the diagnostic process. A standard therapeutic assessment ^18^F-FDG PET/CT (t2-PET) will be performed after the induction sequence and before maintenance initiation (between the 4^th^ and 5^th^ cycles) on the same LAFOV system with an optional WBdyn acquisition. The WBdyn PET imaging protocol is displayed in **Fig 3**. All acquisitions will be performed with a Biograph Vision Quadra^TM^ (Siemens Healthineers©, Knoxville, TN, USA) in the Nuclear Medicine Department (University Hospital of Brest). All PET/CT imaging data will be centralised for analysis. Dynamic PET quantification and kinetic modelling will be performed by a single experienced reader, to ensure a standardised and reproducible analysis pipeline. This approach was chosen to minimise methodological variability in kinetic modelling. As dynamic PET quantification is performed centrally by a single experienced reader in this pilot study, formal interobserver reproducibility assessment is beyond the scope of the present work and will be addressed in future validation studies. All patients will be fasted for at least 6 hours and blood glucose level will be checked prior to ^18^F-FDG administration at a dose of 2 MBq/kg.

The WBDyn acquisition protocol will consist of a 65-minute, one-bed list-mode acquisition of 106 cm. Long dynamic acquisitions may be associated with patient discomfort and motion artefacts. Patient suitability for prolonged acquisition will be assessed upfront (e.g., controlled pain, absence of severe dyspnoea, and ability to remain still), and motion-related non-evaluable scans will be documented. To extract kinetic parameters (Ki, metabolic volume), the FDG blood concentration activity over time (input function) and the voxel concentration activity over time will be required. The Ki metrics will be calculated using the following equation:


$$\frac{C_{V}(t)}{C_{B}(t)}=K_{i}\left(\frac{\int_{\text{0}}^{t}C_{B}(t)}{C_{B}(t)}\right)+D_{V}$$

Where C_V_(t) is the voxel activity concentration over time and C_B_(t) is the blood activity concentration over time. The parametric images, where voxel values correspond to Ki and DV, can be calculated using either a direct reconstruction method (4D Nested iterative reconstruction, Siemens) or an indirect method (OSEM dynamic reconstruction and per-voxel graphic regression, PetKinetX). Both methods will be performed with a standard or an optimized procedure.

The standard procedure will use an image-based input function (IDIF). Direct Patlak analysis using IDIF will be considered the reference method for kinetic quantification. The PET data will be divided into 34 frames over the 60-minute acquisition time (12 images x 5s, 6 images x 10s, 8 images x 30s and 8 images x 5 min), and an automatic VOI will be placed anatomically in the left ventricle to measure the input function. As this procedure is time-consuming, an optimized procedure will be assessed, using a population-based input function (PBIF) built from a large number of resampled IDIFs measured on healthy patients. Then, only a part of the data will be used (30–60 min) to scale the PBIF. An exploratory aim of this pilot study is to assess the feasibility of PBIF-based shortened dynamic protocols. Finally, the last 5 minutes of the acquisition will be used for the standard static SUV reconstruction (**Fig 3**).

Volume of interest (VOI) segmentation of tumour lesions will be performed using MiM software (Cleveland, OH, USA) in order to extract all static and dynamic PET parameters. Target lesion selection on baseline LAFOV PET/CT will follow a pre-specified rule: up to five lesions per patient (maximum two per organ system), including the primary tumour whenever feasible, prioritising the highest-uptake FDG-avid lesions. Lesions will be RECIST-measurable when possible; however, non-measurable FDG-avid lesions (e.g., bone lesions without a clear morphological correlate) may also be included for PET quantitative analyses. Previously irradiated lesions will not be selected as target lesions and will be excluded from quantitative PET analyses.

### Radiomic analysis

Radiomic feature extraction will be performed following a standardised and reproducible workflow. Target lesions will be segmented using predefined rules, and radiomic features will be computed using validated software according to established recommendations. Prior to analysis, features will undergo quality control, including assessment of distributions, exclusion of non-informative features, and reduction of redundancy (e.g., correlation filtering). Radiomics analyses will be considered exploratory and hypothesis-generating, and will primarily focus on effect-size estimation and association with clinical outcomes. Where multiparametric composite modelling is explored, all modelling choices will be pre-specified and reported transparently to support reproducibility, with future external validation planned in subsequent studies.

### Outcomes

Clinical and radiological follow-up will be performed 3 monthly up to 12 months, according to daily clinical practice [22]. This observational study will not influence patient management.

### Primary endpoints

The primary endpoint is the rate of disease progression or death from any cause within one year after treatment initiation. Disease progression will be assessed by the investigators according to RECIST version 1.1 [21].

### Secondary endpoints

The secondary endpoints are listed below:

1. Correlation between quantitative dynamic and radiomic parameters and clinico-histopathological parameters (clinical, biological, histological, genomic).
2. Net Influx Rate (Ki) and Distribution Volume (DV) values based on direct and indirect Patlak methods with both IDIF and PBIF in LAFOV-PET.
3. Target-to-background ratio (TBR), defined as the ratio of the SUV_max_ of the lesions to the SUV_mean_ of healthy liver tissue or bloodpool (background) determined for each reconstruction algorithm.
4. Contrast-to-noise ratio (CNR), indicating the image quality in the lesion.
5. Objective response rate (ORR), defined as the proportion of patients experiencing an objective response (either complete response [CR] or partial response [PR]) as best response to CT-IO according to RECIST 1.1 criteria [21] with iRECIST [23] applied when clinically appropriate in the immunotherapy setting.
6. Metabolic response rate (MRR), defined as the proportion of patients experiencing a metabolic response (either complete metabolic response [CMR] or partial metabolic response [PMR]) as best response to CT-IO according to Positron Emission Tomography Response Criteria in Solid Tumours version 1.0 (PERCIST v1.0) [24] (and iPERCIST [25] when applicable).
7. Overall survival, defined as the time from initiation of CT-IO to death from any cause.
8. Safety and tolerability according to National Cancer Institute Common Terminology Criteria for Adverse Events, version 5.0 [26]; patient reported outcomes according to The European Organisation for Research and Treatment of Cancer Quality of Life Questionnaire Core 30 (QLQ-C30) [27] and EQ-5D-5L [28] (at baseline, 3 months, 6 months, 12 months).

### Data collection and management

- Plans for assessment and collection of outcomes
- Baseline demographic, clinical (including ECOG performance status, smoking status), histopathological, molecular and biological data will be collected. Efficacy and tolerance of systemic therapy and outcome details will be evaluated prospectively. Immune-related adverse events and any treatment modifications (dose delays, interruptions or discontinuations) will be prospectively recorded in the electronic case report form. Palliative radiotherapy (including target site(s), dose and fractionation) will be recorded.
- Imaging evaluation
- Baseline (t1-PET) and post-induction LAFOV PET/CT (t2-PET) imaging data will be analysed. Dates of first response, best objective response and progressive disease will be reported (assessed by LAFOV PET or CT-scan if indicated). Target lesion will be identified as the most avid tumour lesion on the baseline LAFOV-PET according to the PERCIST 1.0 criteria [23]. All the target lesions will be semiautomatically segmented using a spherical volume of interest (VOI). Different imaging parameters will be analysed on a maximum of 5 target lesions, including the primary tumour, with a maximum of two lesions per organ system when feasible, prioritising the most FDG-avid lesions.
  ◦**Radiomics**

#### Standard (1^st^ order).

- Standard uptake value (SUV), measured as the ratio of decay-corrected activity in the VOI to the injected activity per unit body weight;
- SUV_max_ defined as the hottest voxel within the VOI; and SUL_max_ (corrected for lean body-mass);
- SUV_peak_ calculated as the average value of voxels intensities in a spherical region of interest of 1 cm^3^ around the SUV_max_ voxel;
- Metabolic tumor volume (MTV) defined as the total volume (mL) including primary tumor or metastases measured by different methods at the 40% SUV max threshold;
- Total lesion glycolysis (TLG) in grams (g), defined as MTV x SUV_mean_ (automatically calculated as the mean SUV in each VOI).

MTV and TLG will be assessed at baseline only as pre-treatment prognostic biomarkers; no delta-MTV analysis is planned.

- Target-to-background ratio (TBR), defined as the ratio of the lesions’ SUV_max_ and the SUV_mean_ of healthy liver tissue (background) determined for each reconstruction algorithm.
- Contrast-to-noise ratio (CNR), defined as: (lesion SUV_mean_ – background SUV_mean_)/ SD background

#### Textural features (2^nd^ and 3^rd^ order).

- Entropy, Homogeneity, HGZE, LGZE, LRE and SRE.
  ◦**Dynamics**
- Based on a linear regression model of the Patlak plot:
- Net influx rate (Ki) defined as the tumour uptake coefficient (ml/min/100g);
- Distribution volume (DV) defined as the percentage non-trapped tracer in the reversible compartments and the fractional blood volume (%).

Metabolic variation after four cycles of chemo-immunotherapy will be expressed for each target lesion as the percentage change in SUV values between the baseline and the post-induction PET/CT, categorizing the metabolic response (MR) into four classes. A complete metabolic response (CMR) is considered as a complete resolution of FDG uptake within the target lesion; partial metabolic response (PMR), is defined as a decrease greater than or equal to 30% in SUL_max_ of the target lesion; progressive metabolic disease (PMD) is defined as an increase of at least 30% in SUL_max_, or the appearance of a new FDG-avid metastatic lesion; stable metabolic disease (SMD) is between partial response and metabolic progression.

In the specific context of immunotherapy, immune-modified criteria (iRECIST [23] and/or iPERCIST [25] will be applied when clinically appropriate to account for potential unconfirmed progression.

### Data management and confidentiality and dissemination policy

Data will be collected electronically by the investigators using Excel software (Microsoft). Study data will be stored in a computerised database, with confidentiality maintained in accordance with national data protection legislation. All trial documents will be archived and securely stored for 15 years after the end of the study. A final data quality control will be performed before statistical analysis.

## Statistical analyses

Median follow-up will be calculated using the reverse Kaplan–Meier method from the start of chemo-immunotherapy. The primary endpoint is a binary outcome defined as disease progression or death within one year. Analyses related to the primary endpoint will rely on contingency tables and receiver operating characteristic (ROC) curve analyses, with estimation of sensitivity, specificity, and area under the curve (AUC) with 95% confidence intervals. For this primary binary endpoint, p-values will be obtained using Fisher’s exact test.

Progression-free survival (PFS) and overall survival (OS) will be analysed as secondary outcomes and estimated using the Kaplan–Meier method, with exploratory comparisons performed using log-rank tests and Cox proportional hazards models. Hazard ratios will be reported with 95% CIs estimated using the Wald test.

Correlations between PET-LAFOV quantitative parameters and histological, molecular, or biological markers will be estimated using Pearson correlation coefficients with 95% CIs. Associations between quantitative PET parameters and binary markers (presence/absence) will be evaluated by comparing group means using Student’s t-test or Wilcoxon test, as appropriate. The agreements between the different reconstruction methods and the direct IDIF reference method, will be evaluated using Bland and Altman representations and by estimating intraclass correlation coefficients. The agreement between the PET quantitative parameters with parameters resulting from a degraded post-acquisition reconstruction PET SAFOV “like” will be evaluated using Bland and Altman representations and by estimating correlation coefficients intraclass.

Pre-specified exploratory subgroup analyses will be performed according to histological subtype (squamous vs non-squamous NSCLC). Based on pilot-phase findings, an exploratory composite multiparametric signature combining kinetic and radiomic features may be developed to inform future validation studies. No hypothesis testing is planned for secondary or exploratory analyses. Dynamic and radiomic PET parameters will be analysed as separate predefined families.

Tolerance and quality of life will be the subject of a descriptive analysis in terms of frequencies, means, medians, quartiles. Statistical analyses will be performed using R software (version 4.4.2).

A primary interim analysis will be conducted after the inclusion of the first 40 patients and will be limited to descriptive analyses. Final analysis is planned after completion of study.

### Ethics approval and reporting guidelines

This study has obtained ethical approval from the Comité de Protection des Personnes (CPP) Sud-Est VI, France (CPP reference AU 1962; dossier number SI 24.01462.000282; national reference 2024-A00561-46; internal reference 29BRC24.0049). The study protocol is provided as Supporting Information (S1 and S2 Files). The study is reported in accordance with the SPIRIT 2013 guidelines; the completed checklist is available as Supporting Information (S3 File), and the SPIRIT schedule is presented in **Fig 1**. The study was reviewed on July 5, 2024, and received a favourable opinion on July 15, 2024. The study is also registered on ClinicalTrials.gov (NCT06738680). Any important protocol modifications will be submitted for approval to the CPP and the sponsor (University Hospital of Brest). All approved amendments will be dated, versioned, and communicated to participating investigators and updated in the ClinicalTrials.gov registry (NCT06738680).

The protocol, technical and clinical data will be disseminated through conference presentations and peer-reviewed publications.

## Discussion

PROFIL-1 is the first study aiming to prospectively investigate the potential role of dynamic LAFOV-PET in predicting the outcome of first-line CT-IO in patients with metastatic NSCLC without AGA. This study introduces the use of a LAFOV-PET system, that provides dynamic whole-body imaging capabilities with improved sensitivity. This innovative approach allows a more accurate characterization of tumour heterogeneity and metabolic activity compared to conventional PET imaging [11,29]. By integrating multiparametric analysis, including radiomic and dynamic imaging metrics, this study aims to provide a comprehensive evaluation of treatment response and improve the predictive accuracy of CT-IO efficacy.

The primary objective of this study is to evaluate the predictive role of combined standard static and dynamic parameters derived from the whole body ^18^F-FDG PET/CT images at baseline on progression-free survival in metastatic NSCLC patients treated with CT-IO. Indeed, the prediction of response to immunotherapy is currently primarily based on PD-L1 expression on tumour cells, which remains imperfect. PET imaging biomarkers are therefore a promising additional approach to improve patient selection. Variation of conventional first-order radiomic semi-quantitative parameters such as SUV images with static whole-body PET/CT is often considered in the literature as a standard approach for treatment response monitoring, especially in lung cancer [**7**]. Nonetheless, Silvestri et al. had previously highlighted that quantitative parameters identified at the voxel level could characterize the intra-tumoral inhomogeneity in a cohort of 19 patients with NSCLC [29]. Indeed, differences in metabolic activity and vascularization confirmed the wild variability in lung cancers and metastatic lymph nodes. The authors also noted that assessing the spatially heterogeneity of tissues might be relevant to evaluate the response to treatment and to accurately estimate patient prognosis.

We also anticipate that the integration of LAFOV-PET dynamic parameters, such as the tracer influx rate (Ki) and the information on the spatio-temporal distribution of the radiotracer [12], will provide significant insights into the tumour biology of NSCLC, potentially identifying a high-risk subgroup of patients who may require therapeutic escalation. Early identification of non-responders could lead to timely changes in treatment strategies, ultimately improving patient outcomes. WB-dynPET techniques using Ki approach (Patlak analysis), reduce the temporal dependence and the impact of background physiological activity, as found in the liver or mediastinum. The Patlak analysis is a graphical method used to evaluate tracer kinetics and quantify metabolic rates in PET imaging [30]. Ki could therefore become a relevant surrogate marker for SUV, especially as a non-linear Ki/SUV relationship has been suggested [15]. Few data on the topic exist in the literature, published by the same team on small cohorts, while we expect to include 120 patients. Indeed, Wang *et al.* stratified 30 advanced NSCLC patients into a fast dynamic FDG uptake group and a slow dynamic FDG uptake group by unsupervised K-means classification of primary tumours (PTs) using a dynamic acquisition. This stratification led to a more accurate characterization of the homogeneity of PTs in terms of FDG uptake and was correlated with increased immune cell infiltration [31]. They also reported the predictive role of whole body dynamic ^18^F-FDG PET/CT in a cohort of 37 patients with locally advanced NSCLC and treated with two cycles induction of CT-IO [20]. They focused on primary tumours features and stratified patients into high-FDG Patlak-Ki and low-FDG Patlak-Ki groups. Interestingly, while patients could not be accurately classified using standard static PET parameters, those with high FDG-Ki demonstrated better response to induction CT-IO and higher levels of immune cell infiltration in the primary tumours compared to those with low FDG-Ki, demonstrating the dramatic importance of dynamic acquisition.

We will also assess parameters of early response in patients undergoing a second LAFOV PET with optional dynamic acquisition. However, only one study has evaluated the feasibility of using Delta Ki as a criterion for assessing treatment assessment [32].

Recently, several reviews have presented the advantages and potential clinical applications of LAFOV-PET, highlighting its impact across various fields, including oncology [11,33]. Their ability to image the entire body in a single bed position is essential, as its limits the potential motion artifacts associated with multipass acquisition in SAFOV PET [34], the simultaneous evaluation of the kinetics of multiple lesions being primordial for optimal therapeutic assessment in case of metastatic disease, as reported for solid cancer [35]. These PET systems substantially increase detection sensitivity and temporal sampling [36], thus allowing either shorter or low-dose imaging, while maintaining or even improving parametric accuracy [37,38]. With PROFIL-1, we aim to confirm this potential for optimisation of multiparametric analysis in LAFOV PET, including thus the precision of mobile lesions delineation, which is essential for radiomic and kinetic analysis [11].

Among the secondary objectives, we would like to confirm the possibility of using a PBIF to reduce acquisition time in routine clinical practice. Several investigations have introduced shortened protocols to simplify implementation, such as the use of a population-based input function (PBIF) adapted to late dynPET acquisitions on SAFOV PET system. For example, Dias et al. [39] demonstrated that PBIFs can provide reliable input functions with minimal IDIF/PBIF Ki metrics bias in SAFOV PET imaging (<10%). Similarly, Pavoine et al. [14] assessed FDG PET imaging in melanoma patients and found that PBIF approach provided accurate quantification, suggesting their potential as a non-invasive alternative to arterial sampling with less disadvantages than IDIF approach. Indovina et al. [40] and Du et al. [41] further corroborated these findings in lung cancer studies, indicating that PBIF can effectively replace invasive procedures without compromising accuracy. However, these studies also highlighted the need for optimization to reduce bias and shorten acquisition times, which currently range from 20 to 30 minutes. When combined with PBIF-based acquisition protocols, LAFOV scanners can perform near whole-body kinetic assessments over shorter time intervals compared to SAFOV system (up to 10–15 minutes) with similar IDIF vs. PBIF biases. Van Sluis et al. [42] reported that LAFOV PET allows for acquisition times as short as 10–15 minutes without significant loss of data quality. Finally, Palard-Novello et al. [43] demonstrated that this approach is feasible with different tracers, expanding the versatility of LAFOV PET systems. These advances suggest that integrating the PBIF analysis into LAFOV PET imaging could improve non-invasive quantification in oncology and nuclear medicine applications, providing a more efficient alternative to traditional methods.

Another approach of our study is to test an indirect Patlak method as a robust alternative to the direct method. The use of indirect Patlak post-reconstruction tools has been developed, showing equivalent results for shortened scan duration with the PBIF approach, with the added advantage that post-reconstruction is not time consuming for daily practice. Lan et al. [44] demonstrated that indirect Ki estimation using PBIF is feasible with acquisition times as short as 15 minutes, offering another efficient alternative to time-consuming IDIF approach. In this study, we will test a vendor-independent software package developed by Besson et al. [45] (PET KinetiX), which allows very fast calculation of parametric images even with complex compartmental model (e.g., Sokolov) from dynamic PET data.

PROFIL-1 may encounter some limitations, including a relatively small sample size and a short follow-up duration due to the pilot nature of the study. Additionally, the non-interventional design may introduce biases related to patient selection and treatment variability. Moreover, a central independent radiological review is not feasible in this pilot study due to the lack of dedicated funding; therefore, response assessment will rely on predefined criteria (RECIST 1.1/iRECIST and PERCIST/iPERCIST when applicable [21,23–25]) and experienced readers. Due to the single-arm design, the present study will primarily assess prognostic/associative value; evaluation of predictive value will require comparative cohorts or pooled analyses. Retrospective dynamic PET control cohorts are not feasible given the limited availability and non-standardised nature of dynamic LAFOV acquisitions. Future studies with larger cohorts and longer follow-up are needed to externally validate our expected findings and improve generalizability. Dynamic acquisitions with LAFOV PET may present challenges such as increased data volume and the need for complex reconstruction algorithms [46]. Thus, studies have shown that the use of PBIF may not always yield significant differences when compared to IDIF, highlighting the necessity for further optimization [14,47,48].

Our study opens up interesting ways for future research, particularly the integration of a panel of immune markers expressed in immunohistochemistry to evaluate immune cell infiltration in the tumour microenvironment in addition to gene profiling may offer insights into resistance mechanisms and sensitivity to therapy. Moreover, the integration of liquid biopsy to detect ctDNA clearance at different time points during the follow-up and correlation with LAFOV PET multiparametric features may provide a more robust predictive model of CT-IO efficacy and help in monitoring treatment-related toxicities [10].

## Conclusion

The PROFIL-1 study represents a significant step forward in the quest to improve treatment outcomes in advanced NSCLC. By leveraging the capabilities of LAFOV-PET and a multiparametric analytical approach, we hope to enhance the predictive accuracy of CT-IO efficacy and ultimately guide more effective patient management strategies.

### Strengths and limitations of this study

- This is the first prospective study exploring the association between pre-treatment dynamic PET biomarkers and radiomic features with clinical and metabolic response to first-line chemo-immunotherapy in advanced NSCLC.
- The use of a long axial field-of-view PET/CT system allowed full-body dynamic acquisitions with high temporal resolution, enabling robust parametric modelling.
- The methodological approach combined dynamic and radiomic analyses from both primary tumour and metastatic sites, providing a refined assessment of tumour heterogeneity.
- This is a multicentre study with a relatively small sample size, which may limit the generalizability of the results.
- Tumour segmentation was manually performed and, despite validation by experienced nuclear medicine physicians, may be subject to interobserver variability, particularly for radiomic feature extraction.

## Supporting information

S1 File Approved study protocol (English version). Clean English version of the PROFIL-1 study protocol approved by the Comité de Protection des Personnes Sud-Est VI.
(PDF)

S2 File Approved study protocol (French version). Original French version of the PROFIL-1 study protocol approved by the Comité de Protection des Personnes Sud-Est VI.
(PDF)

S3 File SPIRIT checklist. Completed SPIRIT 2013 checklist for the PROFIL-1 study protocol.
(PDF)

## References

[1] Bray F, Laversanne M, Sung H, Ferlay J, Siegel RL, Soerjomataram I. Global cancer statistics 2022: GLOBOCAN estimates of incidence and mortality worldwide for 36 cancers in 185 countries. CA Cancer J Clin. 2024;74(3):229–63. doi: 10.3322/caac.21834 38572751

[2] Allemani C, Matsuda T, Di Carlo V, Harewood R, Matz M, Nikšić M, et al. Global surveillance of trends in cancer survival 2000-14 (CONCORD-3): analysis of individual records for 37 513 025 patients diagnosed with one of 18 cancers from 322 population-based registries in 71 countries. Lancet. 2018;391(10125):1023–75. doi: 10.1016/S0140-6736(17)33326-3 29395269 PMC5879496

[3] Gandhi L, Rodríguez-Abreu D, Gadgeel S, Esteban E, Felip E, De Angelis F, et al. Pembrolizumab plus Chemotherapy in Metastatic Non-Small-Cell Lung Cancer. N Engl J Med. 2018;378(22):2078–92. doi: 10.1056/NEJMoa1801005 29658856

[4] Paz-Ares L, Luft A, Vicente D, Tafreshi A, Gümüş M, Mazières J, et al. Pembrolizumab plus chemotherapy for squamous non-small-cell lung cancer. N Engl J Med. 2018;379(21):2040–51. doi: 10.1056/NEJMoa1810865 30280635

[5] Duchemann B, Remon J, Naigeon M, Cassard L, Jouniaux JM, Boselli L, et al. Current and future biomarkers for outcomes with immunotherapy in non-small cell lung cancer. Transl Lung Cancer Res. 2021;10(6):2937–54. doi: 10.21037/tlcr-20-839 34295689 PMC8264336

[6] Dagogo-Jack I, Shaw AT. Tumour heterogeneity and resistance to cancer therapies. Nat Rev Clin Oncol. 2018;15(2):81–94. doi: 10.1038/nrclinonc.2017.166 29115304

[7] Salaün P-Y, Abgral R, Malard O, Querellou-Lefranc S, Quere G, Wartski M, et al. Good clinical practice recommendations for the use of PET/CT in oncology. Eur J Nucl Med Mol Imaging. 2020;47(1):28–50. doi: 10.1007/s00259-019-04553-8 31637482

[8] Xing X, Zhao Q, Zhou J, Zhou R, Liu Y, Qin X, et al. Positron emission tomography molecular imaging to monitor anti-tumor systemic response for immune checkpoint inhibitor therapy. Eur J Nucl Med Mol Imaging. 2023;50(6):1671–88. doi: 10.1007/s00259-022-06084-1 36622406 PMC10119238

[9] Evangelista L, Fiz F, Laudicella R, Bianconi F, Castello A, Guglielmo P, et al. PET Radiomics and Response to Immunotherapy in Lung Cancer: A Systematic Review of the Literature. Cancers (Basel). 2023;15(12):3258. doi: 10.3390/cancers15123258 37370869 PMC10296704

[10] Hughes DJ, Subesinghe M, Taylor B, Bille A, Spicer J, Papa S, et al. 18F FDG PET/CT and Novel Molecular Imaging for Directing Immunotherapy in Cancer. Radiology. 2022;304(2):246–64. doi: 10.1148/radiol.212481 35762888

[11] Abgral R, Bourhis D, Salaun P-Y. Clinical perspectives for the use of total body PET/CT. Eur J Nucl Med Mol Imaging. 2021;48(6):1712–8. doi: 10.1007/s00259-021-05293-4 33742236

[12] Karakatsanis NA, Lodge MA, Tahari AK, Zhou Y, Wahl RL, Rahmim A. Dynamic whole-body PET parametric imaging: I. Concept, acquisition protocol optimization and clinical application. Phys Med Biol. 2013;58(20):7391–418. doi: 10.1088/0031-9155/58/20/7391 24080962 PMC3941007

[13] Zaidi H, Karakatsanis N. Towards enhanced PET quantification in clinical oncology. Br J Radiol. 2018;91(1081):20170508. doi: 10.1259/bjr.20170508 29164924 PMC6049841

[14] Pavoine M, Thuillier P, Karakatsanis N, Legoupil D, Amrane K, Floch R, et al. Clinical application of a population-based input function (PBIF) for a shortened dynamic whole-body FDG-PET/CT protocol in patients with metastatic melanoma treated by immunotherapy. EJNMMI Phys. 2023;10(1):79. doi: 10.1186/s40658-023-00601-3 38062278 PMC10703763

[15] Thuillier P, Bourhis D, Metges JP, Le Pennec R, Amrane K, Schick U, et al. Prospective study of dynamic whole-body 68Ga-DOTATOC-PET/CT acquisition in patients with well-differentiated neuroendocrine tumors. Sci Rep. 2021;11(1):4727. doi: 10.1038/s41598-021-83965-9 33649421 PMC7921579

[16] Sari H, Teimoorisichani M, Mingels C, Alberts I, Panin V, Bharkhada D, et al. Quantitative evaluation of a deep learning-based framework to generate whole-body attenuation maps using LSO background radiation in long axial FOV PET scanners. Eur J Nucl Med Mol Imaging. 2022;49(13):4490–502. doi: 10.1007/s00259-022-05909-3 35852557 PMC9606046

[17] Hatt M, Tixier F, Pierce L, Kinahan PE, Le Rest CC, Visvikis D. Characterization of PET/CT images using texture analysis: the past, the present… any future?. Eur J Nucl Med Mol Imaging. 2017;44(1):151–65. doi: 10.1007/s00259-016-3427-0 27271051 PMC5283691

[18] Manafi-Farid R, Karamzade-Ziarati N, Vali R, Mottaghy FM, Beheshti M. 2-[18F]FDG PET/CT radiomics in lung cancer: An overview of the technical aspect and its emerging role in management of the disease. Methods. 2021;188:84–97. doi: 10.1016/j.ymeth.2020.05.023 32497604

[19] Reuzé S, Schernberg A, Orlhac F, Sun R, Chargari C, Dercle L, et al. Radiomics in nuclear medicine applied to radiation therapy: methods, pitfalls, and challenges. Int J Radiat Oncol Biol Phys. 2018;102(4):1117–42. doi: 10.1016/j.ijrobp.2018.05.022 30064704

[20] Wang D, Qiu B, Liu Q, Xia L, Liu S, Zheng C, et al. Patlak-Ki derived from ultra-high sensitivity dynamic total body [18F]FDG PET/CT correlates with the response to induction immuno-chemotherapy in locally advanced non-small cell lung cancer patients. Eur J Nucl Med Mol Imaging. 2023;50(11):3400–13. doi: 10.1007/s00259-023-06298-x 37310427

[21] Eisenhauer EA, Therasse P, Bogaerts J, Schwartz LH, Sargent D, Ford R, et al. New response evaluation criteria in solid tumours: revised RECIST guideline (version 1.1). Eur J Cancer. 2009;45(2):228–47. doi: 10.1016/j.ejca.2008.10.026 19097774

[22] Hendriks LE, Kerr KM, Menis J, Mok TS, Nestle U, Passaro A. Non-oncogene-addicted metastatic non-small-cell lung cancer: ESMO Clinical Practice Guideline for diagnosis, treatment and follow-up. Ann Oncol. 2023;34(4):358–76. doi: 10.1016/j.annonc.2022.12.013 36669645

[23] Seymour L, Bogaerts J, Perrone A, Ford R, Schwartz LH, Mandrekar S, et al. iRECIST: guidelines for response criteria for use in trials testing immunotherapeutics. Lancet Oncol. 2017;18(3):e143–52. doi: 10.1016/S1470-2045(17)30074-8 28271869 PMC5648544

[24] Wahl RL, Jacene H, Kasamon Y, Lodge MA. From RECIST to PERCIST: Evolving Considerations for PET Response Criteria in Solid Tumors. J Nucl Med. 2009;50(Suppl 1):122S-50S. doi: 10.2967/jnumed.108.057307 PMC2755245 19403881

[25] Goldfarb L, Duchemann B, Chouahnia K, Zelek L, Soussan M. Monitoring anti-PD-1-based immunotherapy in non-small cell lung cancer with FDG PET: introduction of iPERCIST. EJNMMI Res. 2019;9(1):8. doi: 10.1186/s13550-019-0473-1 30694399 PMC6890907

[26] Common Terminology Criteria for Adverse Events (CTCAE). https://ctep.cancer.gov/protocolDevelopment/electronic_applications/docs/CTCAE_v5_QuickReference_8.5x11.pdf

[27] Aaronson NK, Ahmedzai S, Bergman B, Bullinger M, Cull A, Duez NJ, et al. The European Organization for Research and Treatment of Cancer QLQ-C30: a quality-of-life instrument for use in international clinical trials in oncology. J Natl Cancer Inst. 1993;85(5):365–76. doi: 10.1093/jnci/85.5.365 8433390

[28] EuroQol Research Foundation. EQ-5D-5L User Guide. 2019. https://euroqol.org/publications/user-guides

[29] Silvestri E, Scolozzi V, Rizzo G, Indovina L, Castellaro M, Mattoli MV, et al. The kinetics of 18F-FDG in lung cancer: compartmental models and voxel analysis. EJNMMI Res. 2018;8(1):88. doi: 10.1186/s13550-018-0439-8 30159686 PMC6115323

[30] Patlak CS, Blasberg RG. Graphical evaluation of blood-to-brain transfer constants from multiple-time uptake data. Generalizations. J Cereb Blood Flow Metab. 1985;5(4):584–90. doi: 10.1038/jcbfm.1985.87 4055928

[31] Wang D, Zhang X, Liu H, Qiu B, Liu S, Zheng C, et al. Assessing dynamic metabolic heterogeneity in non-small cell lung cancer patients via ultra-high sensitivity total-body [18F]FDG PET/CT imaging: quantitative analysis of [18F]FDG uptake in primary tumors and metastatic lymph nodes. Eur J Nucl Med Mol Imaging. 2022;49(13):4692–704. doi: 10.1007/s00259-022-05904-8 35819498

[32] Wang D, Mo Y, Liu F, Zheng S, Liu H, Li H, et al. Repeated dynamic [18F]FDG PET/CT imaging using a high-sensitivity PET/CT scanner for assessing non-small cell lung cancer patients undergoing induction immuno-chemotherapy followed by hypo-fractionated chemoradiotherapy and consolidative immunotherapy: report from a prospective observational study (GASTO-1067). Eur J Nucl Med Mol Imaging. 2024;51(13):4083–98. doi: 10.1007/s00259-024-06819-2 38953934

[33] Mohr P, van Sluis J, Providência L, van Snick JH, Lub-de Hooge MN, Willemsen AT. Long versus short axial field of view immuno-PET/CT: semiquantitative evaluation for 89Zr-trastuzumab. J Nucl Med. 2023;64(11):1815–20. doi: 10.2967/jnumed.123.265621 37536740

[34] Kaji T, Osanai K, Nakata T, Tamaki N. Dynamic whole-body 18F-FDG PET for minimizing patient motion artifact. Clin Nucl Med. 2020;45(11):880–2. doi: 10.1097/RLU.0000000000003253 32969898

[35] Alberts I, Seibel S, Xue S, Viscione M, Mingels C, Sari H, et al. Investigating the influence of long-axial versus short-axial field of view PET/CT on stage migration in lymphoma and non-small cell lung cancer. Nucl Med Commun. 2023;44(11):988–96. doi: 10.1097/MNM.0000000000001745 37578376 PMC10566597

[36] Alberts I, Hünermund J-N, Prenosil G, Mingels C, Bohn KP, Viscione M, et al. Clinical performance of long axial field of view PET/CT: a head-to-head intra-individual comparison of the Biograph Vision Quadra with the Biograph Vision PET/CT. Eur J Nucl Med Mol Imaging. 2021;48(8):2395–404. doi: 10.1007/s00259-021-05282-7 33797596 PMC8241747

[37] Liu G, Hu P, Yu H, Tan H, Zhang Y, Yin H, et al. Ultra-low-activity total-body dynamic PET imaging allows equal performance to full-activity PET imaging for investigating kinetic metrics of 18F-FDG in healthy volunteers. Eur J Nucl Med Mol Imaging. 2021;48(8):2373–83. doi: 10.1007/s00259-020-05173-3 33479842

[38] Pedersen MA, Dias AH, Hjorthaug K, Gormsen LC, Fledelius J, Johnsson AL, et al. Increased lesion detectability in patients with locally advanced breast cancer - A pilot study using dynamic whole-body [18F]FDG PET/CT. EJNMMI Research. 2024;14(1):31. doi: 10.1186/s13550-024-01096-4 38528239 PMC10963357

[39] Dias AH, Smith AM, Shah V, Pigg D, Gormsen LC, Munk OL. Clinical validation of a population-based input function for 20-min dynamic whole-body 18F-FDG multiparametric PET imaging. EJNMMI Phys. 2022;9(1):60. doi: 10.1186/s40658-022-00490-y 36076097 PMC9458803

[40] Indovina L, Scolozzi V, Capotosti A, Sestini S, Taralli S, Cusumano D, et al. Short 2-[18F]Fluoro-2-Deoxy-D-Glucose PET dynamic acquisition protocol to evaluate the influx rate constant by regional Patlak graphical analysis in patients with non-small-cell lung cancer. Front Med (Lausanne). 2021;8:725387. doi: 10.3389/fmed.2021.725387 34881253 PMC8647994

[41] Du F, Wumener X, Zhang Y, Zhang M, Zhao J, Zhou J, et al. Clinical feasibility study of early 30-minute dynamic FDG-PET scanning protocol for patients with lung lesions. EJNMMI Phys. 2024;11(1):23. doi: 10.1186/s40658-024-00625-3 38441830 PMC10914647

[42] van Sluis J, van Snick JH, Glaudemans AWJM, Slart RHJA, Noordzij W, Brouwers AH. Ultrashort oncologic whole-body [18F]FDG Patlak imaging using LAFOV PET. J Nucl Med. 2024;65(10):1652–7. doi: 10.2967/jnumed.124.267784 39353647

[43] Palard-Novello X, Visser D, Tolboom N, Smith CLC, Zwezerijnen G, van de Giessen E, et al. Validation of image-derived input function using a long axial field of view PET/CT scanner for two different tracers. EJNMMI Phys. 2024;11(1):25. doi: 10.1186/s40658-024-00628-0 38472680 PMC10933214

[44] Lan W, Sari H, Rominger A, Fougère C la, Schmidt FP. Optimization and impact of sensitivity mode on abbreviated scan protocols with population-based input function for parametric imaging of [18F]-FDG for a long axial FOV PET scanner. Eur J Nucl Med Mol Imaging. 2024;51(11):3346–59. doi: 10.1007/s00259-024-06745-3 38763962 PMC11368996

[45] Besson FL, Faure S. PET KinetiX-A Software Solution for PET Parametric Imaging at the Whole Field of View Level. J Imaging Inform Med. 2024;37(2):842–50. doi: 10.1007/s10278-023-00965-z 38343229 PMC11031504

[46] Li Y, Hu J, Sari H, Xue S, Ma R, Kandarpa S, et al. A deep neural network for parametric image reconstruction on a large axial field-of-view PET. Eur J Nucl Med Mol Imaging. 2023;50(3):701–14. doi: 10.1007/s00259-022-06003-4 36326869

[47] Naganawa M, Gallezot JD, Shah V, Mulnix T, Young C, Dias M. Assessment of population-based input functions for Patlak imaging of whole body dynamic 18F-FDG PET. EJNMMI Phys. 2020;7(1):67. doi: 10.1186/s40658-020-00330-x 33226522 PMC7683759

[48] Thuillier P, Bourhis D, Pavoine M, Metges J-P, Le Pennec R, Schick U, et al. Population-based input function (PBIF) applied to dynamic whole-body 68Ga-DOTATOC-PET/CT acquisition. Front Nucl Med. 2022;2:941848. doi: 10.3389/fnume.2022.941848 39390995 PMC11464975

