# Supplementary material for: PRognostic and predictive potential Of multiparametric dynamic whole-body 18F-FDG PET Imaging using a Long axial field-of-view (LAFOV) system for FIRST-line chemo-immunotherapy efficacy in advanced non-small cell lung cancer: PROFIL-1 study protocol
Source: PLoS One. 2026 Apr 3;21(4):e0345990. doi: 10.1371/journal.pone.0345990 (PMC13048445; doi:10.1371/journal.pone.0345990)
Supplement: S1 File — Clean English version of the PROFIL-1 study protocol approved by the Comité de Protection des Personnes Sud-Est VI. (PDF) [file pone.0345990.s001.pdf]

**SPONSOR**	University Hospital of Brest (CHU de Brest)
**CATEGORY 3 HUMAN RESEARCH PROTOCOL**	
**ACRONYM**<br><br>**STUDY CODE**<br><br>**IDRCB NUMBER**	**PROFIL-1**<br><br>29BRC24.0049<br><br>**2024-A00561-46**
**FULL TITLE**<br><br>**INDICATION (TARGET)**	**PRedictive value Of multiparametric dynamic whole-body FDG-PET Imaging on a LAFOV system for first-line chemo-immunotherapy efficacy in advanced non-small-cell lung cancer (NSCLC) (PROFIL-1)**<br><br>Non-Small Cell Lung Cancer (NSCLC)
**COORDINATING INVESTIGATORS:**	**Dr Margaux GEIER**<br>Department of Medical<br>Oncology, University Hospital<br>of Brest<br>Tel: +33 (0)2 30 33 80 30<br>**Pr Ronan ABGRAL**<br>Department of Nuclear Medicine, University Hospital of Brest
**PROTOCOL VERSION NUMBER**	V1.0
**PROTOCOL DATE**	14 March 2024
**ETHICS APPROVAL**	Approved on 15 July 2024<br>by the Comité de Protection des Personnes (CPP) Sud-Est VI
**CONFIDENTIALITY STATEMENT:**<br>**This confidential document is the property of the University Hospital of Brest. No unpublished information contained herein may be disclosed without the prior written authorisation of the University Hospital of Brest.**	

Amendment History

VERSION NUMBER (AFTER AMENDMENT)	DATE	RATIONALE FOR AMENDMENT

SIGNATURES

SIGNATURE OF THE SPONSOR

NAME : University Hospital of Brest (CHU Brest)

Signature : CHU Brest Date : 15 July 2024

SIGNATURE OF THE COORDINATING INVESTIGATOR

I have read all pages of the research protocol for which the University Hospital of Brest is the sponsor.

I confirm that it contains all the information necessary for the proper conduct of the study.

I undertake to carry out the study in accordance with the protocol and the terms and conditions defined therein.

I further commit to conducting the study in compliance with:

the principles of the Declaration of Helsinki;

the international (ICH-E6) and French Good Clinical Practice (GCP) guidelines applicable to research involving human participants;

the national legislation and regulations governing research involving human participants (RIPH).

I also undertake to ensure that all investigators and other qualified members of my team have access to copies of this protocol and all study-related documents necessary to perform their tasks in compliance with the provisions contained therein.

NOM: GEIER Margaux

Signature : M Geier Date : 15 July 2024

SIGNATURE OF THE PRINCIPAL INVESTIGATOR AT THE ASSOCIATED SITE

NOM : ________________________ Centre : ____________

Signature : ……………………………………………… Date : ____________

TABLE OF CONTENTS

SIGNATURES 3
SPONSOR'S SIGNATURE 3
- Title 5
- Sponsor 5
- Coordinators 5
- Principal Co-Investigators 5
- Associated Investigators 5
- Study Coordination and Monitoring 6
- Associated Scientists 6
- Methodologist – Biostatistician 6
2. SCIENTIFIC RATIONALE AND GENERAL DESCRIPTION OF THE STUDY 11
2.1. Designation and description of the disease 11
2.2. Study background and literature review 11
2.3 Summary of relevant previous studies 12
2.4. Justification of the scientific relevance of the study 12
3. OBJECTIVES 12
3.1. Primary objective 12
3.2. Secondary objectives 12
4. STUDY DESIGN AND CONDUCT 13
4.1. Study qualification and investigator credentials. 13
4.2. Evaluation criteria 13
4.3. Study procedures 14
Inclusion period: 2 years 15
Patient participation period: 1 year 15
Overall study duration: 3 years 15
5. STUDY POPULATION 15
5.1. Description of the source population 15
5.2 Inclusion and non-inclusion criteria 15
5.3 Feasibility 15
6. RECRUITMENT PROCEDURES AND PARTICIPANT INFORMATION
15
7. STATISTICS 16
7.1. Statistical lead and data storage site 16
7.2. Sample size justification 16
7.3. Statistical analysis methods 16
7.4 Handling of missing, unused, or invalid data 17
7.5 Selection of participants included in the analysis 17
8. DATA HANDLING, STORAGE, AND RETENTION 17
8.1. Case report form 17
8.2. Identification of data recorded in the case report form 18
8.4. CNIL declaration 19
8.5. Data processing 20
8.6. Data archiving 20
9. ETHICAL AND LEGAL ASPECTS 20
9.1. Legal obligations. 20
9.2. Protocol amendments 21
9.3. Final study summary 21
10. PUBLICATION AND DATA OWNERSHIP 21
11. REFERENCES 22

General informations :

- **Title:**

  PRedictive value **O**f multiparametric dynamic whole-body **F**DG-PET **I**maging on a **L**AFOV system for **first**-line chemo-immunotherapy efficacy in advanced non-small-cell lung cancer (NSCLC) (**PROFIL-1**)

  Valeur prédictive d'une imagerie dynamique multiparamétrique TEP-FDG corps entier sur système LAFOV de l'efficacité d'une chimio-immunothérapie de 1ère ligne dans le cancer bronchique non à petites cellules (CBNPC) de stade avancé (**PROFIL-1**)

- ***Sponsor :***

  University Hospital of Brest (CHU de Brest)
  2 avenue Foch-29609 Brest cedex

- ***Coordinators :***

  - Dr Margaux GEIER – Onco-pneumologue
    Service d'Oncologie Médicale, CHU MORVAN, 2 avenue FOCH, 29 200 Brest
    margaux.geier@chu-brest.fr
    02 0 33 80 30

- ***Principal Co-Investigators***

  - Pr Ronan ABGRAL
    Service de Médecine Nucléaire CHU Brest, 2 avenue FOCH, 29 200 Brest
    ronan.abgral@chu-brest.fr
    02 98 22 30 69
  - Dr Karim AMRANE (Oncologie CH Morlaix)
    KAmrane@ch-morlaix.fr
    02 98 62 60 38

- ***Associated Investigators***

  - Dr François LUCIA (Radiothérapie CHU Brest)
    francois.lucia@chu-brest.fr
    02 98 23 81 57

  - Dr Vincent BOURBONNE (Radiothérapie CHU Brest)
    vincent.bourbonne@chu-brest.fr
    02 29 02 02 89

  - Pr Pierre-Yves LE ROUX (Médecine Nucléaire CHU Brest)
    pierre-yves.leroux@chu-brest.fr
    02 98 22 33 27

  - Pr Pierre-Yves SALAUN (Médecine Nucléaire CHU Brest)
    pierre-yves.salaun@chu-brest.fr
    02 98 22 33 27

- David BOURHIS (Radiophysicien, Médecine Nucléaire CHU Brest)
  David.bourhis@chu-brest.fr
  02 98 22 30 50

- Dr Renaud DESCOURT (Oncologie Médicale, CHU Brest)
  renaud.descourt@chu-brest.fr
  02 98 22 37 40

- Dr Gilles QUERE (Oncologie Médicale, CHU Brest)
  gilles.quere@chu-brest.fr
  02 98 22 37 40

- Dr Jessica NGUYEN (Oncologie Médicale, CHU Brest)
  jessica.nguyen@chu-brest.fr
  02 98 22 37 40

- ***Study Coordination and Monitoring***

Direction de la Recherche Clinique et de l'Innovation (DRCI)
Hôpital Morvan – 2 avenue Foch
29 609 Brest cedex

- ***Associated Scientists***

- Pr COUTURAUD Francis (Pneumologie, CHU Brest)
  francis.couturaud@chu-brest.fr
  02 98 34 78 26

- Dr Nicolas KARAKATSANIS (Assistant Professor of Biomedical Engineering in Radiology, Weill Cornell Medicine, Cornell University, New York)
  nak2032@med.cornell.edu

- Dr Florent BESSON (Département de Médecine Nucléaire CH Bicêtre, Université Paris Saclay, UMR Inserm BIOMAPS, France)

- ***Methodologist – Biostatistician***

Unité bio statistique – DRCI de Brest

1. SUMMARY

ENGLISH TITLE	**PR**edictive value **O**f multiparametric dynamic whole-body **F**DG-PET **I**maging on a **L**AFOV system for **first**-line chemo-immunotherapy efficacy in advanced non-small-cell lung cancer (NSCLC) (**PROFIL-1**)
FRENCH TITLE	Valeur prédictive d'une imagerie dynamique multiparamétrique TEP-FDG corps entier sur système LAFOV de l'efficacité d'une chimio-immunothérapie de 1ère ligne dans le cancer bronchique non à petites cellules (CBNPC) de stade avancé (**PROFIL-1**)
SPONSOR	University Hospital of Brest
COORDINATOR	Dr Margaux GEIER (Department of Medical Oncology, CHU Brest)
ACRONYM	**PROFIL-1**
RATIONALE FOR THE STUDY	Lung cancer is the leading cause of cancer-related mortality in France and worldwide, with an overall 5-year relative survival rate of approximately 20% across all stages. In around 70% of cases, the disease is diagnosed at an advanced metastatic stage, not amenable to curative treatment.<br><br>The advent of immunotherapy, particularly immune checkpoint inhibitors (ICI), has provided an opportunity to improve outcomes for patients with non-small cell lung cancer (NSCLC). More recently, the combination of chemotherapy and immunotherapy has been validated as the standard first-line treatment for metastatic NSCLC.<br><br>NSCLC exhibits substantial inter- and intra-tumour heterogeneity, which may partly explain differences in treatment response rates and duration. 18F-FDG PET/CT is a functional imaging modality currently recommended for therapeutic assessment of metastatic NSCLC under systemic therapy. The long axial field-of-view PET (LAFOV PET) system is an innovative, state-of-the-art imaging technology that enables improved characterisation of tumour heterogeneity. It offers two major advantages over conventional short axial field-of-view (SAFOV) systems: enhanced detection sensitivity and the ability to perform dynamic whole-body, multi-organ analyses.<br><br>The identification of PET/CT imaging biomarkers to predict early treatment response represents a key challenge in oncology and a critical step towards precision medicine.<br>Our hypothesis is that ^18F-FDG LAFOV PET imaging can improve the prediction of first-line chemo-immunotherapy efficacy in patients with advanced NSCLC, complementing conventional clinical and biological biomarkers.
STUDY POPULATION	Patients diagnosed with advanced-stage non-small cell lung cancer (NSCLC) for whom first-line chemo-immunotherapy has been selected as the indicated treatment.
PRIMARY OBJECTIVE	To evaluate the prognostic performance of a whole-body multiparametric analysis (radiomic and dynamic) using LAFOV-

	PET imaging for assessing progression-free survival (PFS) in a cohort of patients with advanced-stage non-small cell lung cancer (NSCLC) treated with first-line chemo-immunotherapy.
SECONDARY OBJECTIVES	A1. To assess the association between quantitative 18F-FDG LAFOV PET parameters and tumour histological markers (squamous vs non-squamous histology, PD-L1 status [negative, 1–49%, ≥50%], Ki67, VEGF, TILs, and other relevant markers of interest).<br><br>A2. To investigate the correlation between quantitative 18F-FDG LAFOV PET parameters and tumour molecular biomarkers (KRAS, BRAF, HER2, MET, NTRK, NRG1, KEAP1, NFE2L2, STK11, TP53, SMARCA4, and other relevant markers of interest).<br><br>A3. To explore the correlation between quantitative 18F-FDG LAFOV PET parameters and patient biological parameters (neutrophil and lymphocyte counts, LIPI score, eosinophil count, C-reactive protein [CRP], lactate dehydrogenase [LDH], and albumin levels).<br><br>B. To compare direct and indirect Patlak reconstruction methods using PBIF or IDIF approaches for the calculation of dynamic parameters (Ki and DV) on 18F-FDG LAFOV PET.<br><br>C. To compare quantitative parameters derived from 18F-FDG LAFOV PET acquisition with those obtained from post-acquisition degraded "SAFOV-like" reconstructions.<br><br>D. To evaluate additional efficacy outcomes of first-line chemo-immunotherapy, including overall survival (OS) and objective response rate (ORR).<br><br>E. To assess the correlation between quantitative 18F-FDG LAFOV PET parameters and the occurrence of treatment-related toxicity.
PRIMARY ENDPOINT	1 year progression-free survival

SECONDARY ENDPOINTS	A1. Quantitative 18F-FDG LAFOV PET imaging parameters and tumour histological markers (squamous vs non-squamous histology, PD-L1 status [negative, 1–49%, ≥50%], Ki67, VEGF, TILs, and other relevant markers of interest).<br><br>A2. Quantitative 18F-FDG LAFOV PET imaging parameters and tumour molecular biomarkers (KRAS, BRAF, HER2, MET, NTRK, NRG1, KEAP1, NFE2L2, STK11, TP53, SMARCA4, and other relevant markers of interest).<br><br>A3. Quantitative 18F-FDG LAFOV PET imaging parameters and biological parameters (neutrophil and lymphocyte counts, LIPI score, eosinophil count, C-reactive protein [CRP], lactate dehydrogenase [LDH], and albumin levels).<br><br>B. Dynamic parameters (Ki and DV) obtained from direct and indirect Patlak reconstructions using image-derived input function (IDIF) or population-based input function (PBIF).<br><br>C. Number of tumour lesions and signal-to-noise ratio.<br><br>D. Predictive value for overall survival (OS), defined as the time from treatment initiation to death from any cause, and for objective response rate (ORR) assessed according to RECIST and/or PERCIST criteria.<br><br>E. Safety and tolerability (adverse events graded according to CTCAE v5.0), and quality-of-life scores (EQ-5D-5L and EORTC QLQ-C30 questionnaires assessed at baseline, 3 months, 6 months, and 1 year)
METHODS	Prospective, non-interventional, multicentre pilot study (University Hospital of Brest and Morlaix Hospital).
STATISTICS	The objective is to establish relevant threshold values for each parameter using receiver operating characteristic (ROC) curve analysis, and to calculate the corresponding sensitivity and specificity values.<br>With a cohort of 120 patients, of whom approximately 60% are expected to experience tumour progression at one year, it will be possible to estimate sensitivity and specificity with a precision (half-width of the 95% confidence interval) of approximately 10% for sensitivity and 12% for specificity, assuming observed values around 80%.

INCLUSION CRITERIA	- Adult patient aged ≥ 18 years<br>- Diagnosed with advanced, unresectable, non-irradiable, or metastatic non-small cell lung cancer (NSCLC)<br>- Treatment-naïve<br>- Eligible for first-line chemo-immunotherapy including an anti–PD-1 agent<br>- Has provided non-opposition to participation in the study (in accordance with French regulations for non-interventional research)<br>- Eligible for 18F-FDG LAFOV PET/CT imaging to be performed within 21 days prior to treatment initiation
EXCLUSION CRITERIA	- Minor patient (<18 years of age)<br>- Presence of a targetable oncogenic driver alteration eligible for first-line targeted therapy (EGFR, ALK, ROS1, RET)<br>- Pregnancy or breastfeeding<br>- Histology other than non-small cell lung cancer (NSCLC)<br>- Not eligible for first-line chemo-immunotherapy<br>- Not eligible for LAFOV PET/CT imaging<br>- Refusal to participate in the study
NUMBER OF PATIENTS	The final estimated sample size is 120 patients.
STUDY TIMELINE	Inclusion period: 2 years<br>Patient participation duration: 1 year<br>Total study duration: 3 years

EXPECTED OUTCOMES	Non-small cell lung cancer (NSCLC) remains a disease with a poor prognosis, largely because most lesions are metastatic at the time of diagnosis. Despite the availability of various therapeutic regimens and the significant advances achieved with the advent of immunotherapy, NSCLC continues to represent a major public health concern, owing in particular to marked inter- and intra-tumoural heterogeneity in treatment response.<br><br>The early identification of patients who are refractory to treatment would offer a clear advantage, both in terms of improving survival outcomes and optimising healthcare resource utilisation. Despite careful evaluation of known prognostic factors, it remains difficult to reliably predict patient survival.<br><br>The identification of new pre-therapeutic PET/CT parameters, such as the spatio-temporal distribution of the tracer (combining radiomic and kinetic approaches), could therefore provide valuable insights for therapeutic decision-making. The LAFOV PET system, of which only a few units are currently installed worldwide (the first in mainland France), appears to be a promising tool in this context.<br><br>This approach could enable the identification of a subgroup of patients with poor prognosis who might benefit from treatment escalation, either through systemic therapy intensification with additional agents or through stereotactic treatment of oligometastatic refractory lesions.

2. SCIENTIFIC RATIONALE AND GENERAL DESCRIPTION OF THE STUDY

2.1. Designation and Description of the Disease

Lung cancer remains the leading cause of cancer-related mortality in men, with an overall 5-year relative survival rate of approximately 20% across all stages (1). In nearly 70% of cases, the disease is diagnosed at an advanced stage that is not amenable to curative treatment (2,3).

Recent advances in immunotherapy, particularly immune checkpoint inhibitors (ICIs), have provided new opportunities to improve outcomes for patients with non-small cell lung cancer (NSCLC), achieving response rates of around 20% (4–6). More recently, the combination of chemotherapy and immunotherapy has been validated as a first-line treatment for metastatic NSCLC (7,8). However, despite improvements in response duration and long-term survival (9,10), a significant proportion of patients do not benefit from these new anticancer therapies, and reliable predictive markers of response are still lacking.

NSCLC exhibits substantial inter- and intra-tumoural heterogeneity, characterised by cell clones with variable proliferation rates and tumour regions displaying differing degrees of angiogenesis, hypoxia, necrosis, or fibrosis (11). This heterogeneity may partly explain the variability in treatment response. Characterising this tumoural heterogeneity has therefore become a major challenge in oncology, with the goal of optimising therapeutic strategies and moving toward a personalised, patient-specific approach to treatment (12).

2.2. Study Background and Literature Review

18F-FDG PET/CT is a functional imaging modality that characterises the glucose metabolism of tumours and is currently indicated for the therapeutic assessment of metastatic lung cancer under systemic treatment (13). The identification of surrogate imaging biomarkers (“surrogate markers”) on PET/CT to better characterise tumour heterogeneity and to enable early prediction of survival and treatment response remains a major challenge. Long axial field-of-view (LAFOV) PET systems represent an innovative, next-generation imaging technology (fewer than 15 systems currently available in Europe, and only one in France). They offer two major advantages over conventional short axial field-of-view (SAFOV) systems: (i) a substantial gain in detection sensitivity, and (ii) the ability to perform dynamic, whole-body, multi-organ analyses (14). These features open promising avenues for optimising multiparametric PET image analysis. Textural (radiomic) analysis of PET imaging, which quantifies the spatial distribution of voxel intensities, allows for the computation of multiple indices reflecting tumour heterogeneity (15). Several studies have demonstrated the prognostic value of textural analysis for NSCLC using FDG-PET imaging (16). However, the reliability of textural analysis depends on image noise (and therefore on the intrinsic performance of the scanner) and remains limited for small or moving lesions, such as those located in the lungs (17). Four-dimensional dynamic PET (4D dynPET) analysis has been proposed to extract quantitative parameters from the temporal distribution of the radiotracer within voxels (18,19). This approach allows the estimation of kinetic parameters, typically using a Patlak-like modelling method, based on an image-derived input function (IDIF) or a population-based input function (PBIF) (20). Initial studies using dynPET acquisitions have shown a lack of linear correlation between SUV and Ki values (21), suggesting that kinetic data provide additional quantitative information, offering new perspectives for prognostic evaluation in NSCLC (22). LAFOV PET technology has the potential to overcome these current limitations, enabling high-resolution, low-noise image reconstruction and excellent temporal sampling (whole-body acquisition in 10–20 seconds). These capabilities could optimise radiomic analysis and 4D dynamic quantification, thus enhancing the predictive value of PET imaging in NSCLC.

2.3 Summary of Relevant Studies and Rationale for the Proposed Research

A single recent study has demonstrated the potential value of multiparametric analysis using LAFOV PET imaging for predicting response to chemo-immunotherapy in NSCLC. However, that study was limited to primary tumour (T) analysis in locally advanced cases undergoing induction systemic therapy (23).

2.4. Scientific Rationale and Significance of the Study

Non-small cell lung cancer (NSCLC) remains a disease with poor prognosis, largely due to the high proportion of patients diagnosed with metastatic disease at presentation. Despite the availability of various therapeutic regimens and the significant progress achieved with the advent of immunotherapy, NSCLC continues to represent a major public health challenge, in particular because of marked inter- and intra-tumoural heterogeneity in treatment response.

The early identification of patients who are refractory to therapy would offer a clear advantage, not only in improving survival outcomes but also in optimising healthcare resource utilisation. Despite careful evaluation of known prognostic factors, it remains difficult to reliably predict patient survival. The identification of novel pre-therapeutic PET/CT parameters — such as the spatio-temporal distribution of the tracer (combining radiomic and kinetic approaches) — could be of major interest in guiding therapeutic strategies.

The LAFOV PET system, of which only a few units are currently installed worldwide, appears to have the potential to meet this objective. Identifying a subgroup of patients with poor prognosis could make it possible to consider treatment escalation, either through systemic therapy intensification by adding other agents (24) or through stereotactic treatment of oligometastatic refractory lesions

3. OBJECTIVES

3.1. Primary Objective

To evaluate the prognostic performance of whole-body multiparametric (radiomic and dynamic) 18F-FDG LAFOV PET analysis for assessing progression-free survival (PFS) in a cohort of patients with advanced-stage non-small cell lung cancer (NSCLC) treated with first-line chemo-immunotherapy.

3.2. Secondary Objectives

A1. To assess the association between quantitative 18F-FDG LAFOV PET parameters and tumour histological markers (squamous vs non-squamous histology, PD-L1 status [negative, 1–49%, ≥50%], Ki67, VEGF, tumour-infiltrating lymphocytes [TILs], and other relevant markers of interest).

A2. To investigate the correlation between quantitative 18F-FDG LAFOV PET parameters and tumour molecular biomarkers (KRAS, BRAF, HER2, MET, NTRK, NRG1, KEAP1, NFE2L2, STK11, TP53, SMARCA4, and other relevant markers of interest).

A3. To explore the correlation between quantitative 18F-FDG LAFOV PET parameters and patient biological parameters (neutrophil and lymphocyte counts, LIPI score, eosinophil count, C-reactive protein [CRP], lactate dehydrogenase [LDH], and albumin levels).

B. To compare direct (Siemens) and indirect (PETkinetic) Patlak reconstruction methods using population-based input function (PBIF) or image-derived input function (IDIF) for the calculation of dynamic parameters Ki and DV on 18F-FDG LAFOV PET.

C. To compare quantitative parameters derived from 18F-FDG LAFOV PET acquisition with those

obtained from post-acquisition degraded “SAFOV-like” PET reconstructions.

D. To evaluate additional efficacy outcomes of first-line chemo-immunotherapy, including overall survival (OS) and objective response rate (ORR).

E. To assess the correlation between quantitative 18F-FDG LAFOV PET parameters and the occurrence of treatment-related toxicity.

4. STUDY DESIGN AND CONDUCT

4.1. Study Classification and Investigator Qualifications

This is a multicentre, prospective, non-interventional pilot study conducted in humans with the aim of advancing biological and medical knowledge. All procedures are performed and all products are used in their usual manner, without any additional or unusual diagnostic, therapeutic, or monitoring interventions. The management of patients with non-small cell lung cancer (NSCLC) falls within the recognised field of expertise of the thoracic oncology investigators participating in the study. Nuclear medicine imaging procedures are performed by investigators with established expertise in the Department of Nuclear Medicine.

4.2. Evaluation Criteria

4.2.1. Primary Endpoint

Progression-free survival (PFS), defined as the time interval between the start of first-line chemo-immunotherapy and the date of disease progression or death from any cause, whichever occurs first.

The 1-year progression-free survival (PFS) will be used as the primary endpoint to assess the prognostic performance of 18F-FDG LAFOV PET imaging.

Tumour progression will be evaluated according to RECIST criteria (25) on follow-up CT scans and/or PERCIST criteria (26) when 18F-FDG PET is performed.

4.2.2. Secondary Endpoints

A1. Quantitative 18F-FDG LAFOV PET imaging parameters and histological tumour markers (squamous vs non-squamous histology, PD-L1 status [negative, 1–49%, ≥50%], Ki67, VEGF, TILs, and other relevant markers).

A2. Quantitative 18F-FDG LAFOV PET imaging parameters and molecular tumour biomarkers (KRAS, BRAF, HER2, MET, NTRK, NRG1, KEAP1, NFE2L2, STK11, TP53, SMARCA4, and other relevant markers).

A3. Quantitative 18F-FDG LAFOV PET imaging parameters and biological blood parameters (neutrophil and lymphocyte counts, LIPI score, eosinophil count, CRP, LDH, and albumin levels).

B. Comparison of direct (Siemens) and indirect (PETkinetic) Patlak reconstruction methods using PBIF or IDIF for the calculation of dynamic parameters Ki and DV.

C. Comparison between quantitative parameters from 18F-FDG LAFOV PET acquisition and those from post-acquisition degraded “SAFOV-like” PET reconstruction.

D. Evaluation of additional efficacy endpoints, including overall survival (OS) and objective response rate (ORR) according to RECIST and/or PERCIST criteria.

E. Assessment of the relationship between quantitative 18F-FDG LAFOV PET parameters and treatment-related toxicity (adverse events graded according to CTCAE v5.0; quality-of-life scores EQ-5D-5L and EORTC QLQ-C30 assessed at baseline, 3 months, 6 months, and 12 months).

4.3. Study Procedures

Study design

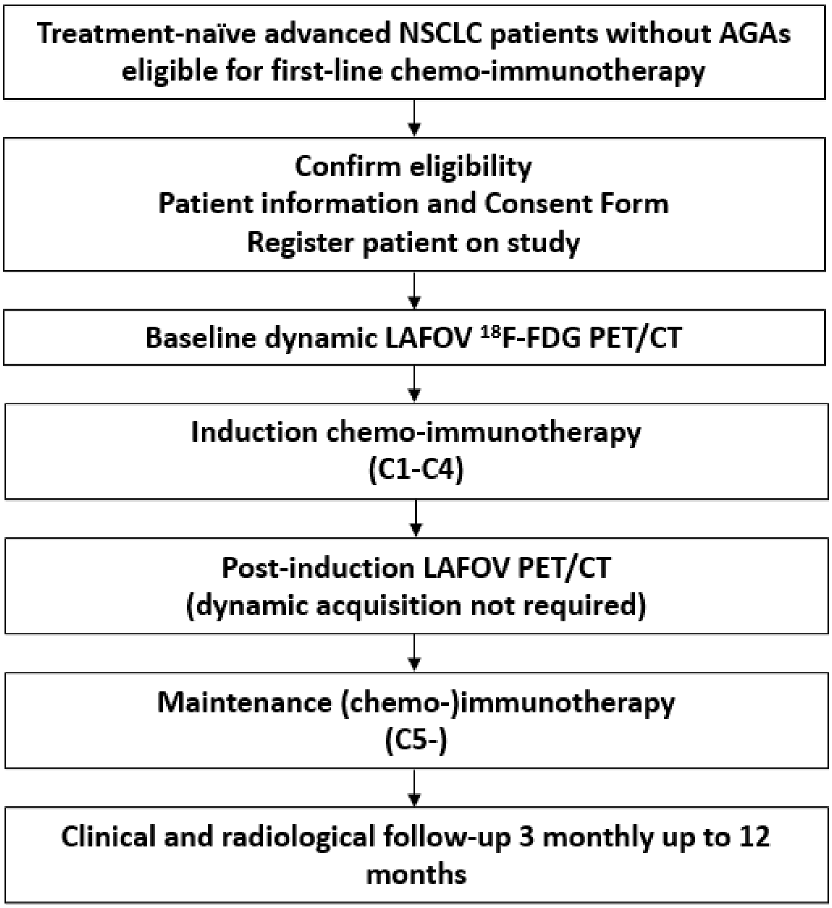

Recruitment Procedures

Recruitment will be conducted prospectively, following approval by the Ethics Committee (Comité de Protection des Personnes), by one of the designated investigators. Eligible patients will be identified during the initial thoracic oncology consultation at the University Hospital of Brest and the Hospital of Morlaix. These will be patients with advanced-stage non-small cell lung cancer (NSCLC) who are eligible to receive standard first-line chemo-immunotherapy.

The inclusion and non-inclusion criteria will be verified, and the patient's non-opposition will be obtained during this consultation through a written consent form. The form will provide complete, fair, and comprehensible information about the study. It will clearly explain the study objectives, the patient's right to refuse participation, and their right to withdraw at any time without consequence. If the patient agrees during the consultation, inclusion will proceed. In case of refusal or no response, the patient will not be included.

A baseline 18F-FDG LAFOV PET/CT will be performed within 21 days prior to the initiation of chemo-immunotherapy. This imaging examination will be considered part of the standard pre-

treatment imaging workup.

Patients will subsequently undergo routine oncological follow-up according to standard clinical practice.

Prospective data collection will include clinical and biological patient characteristics, tumour-related features, and imaging data. All data will be entered into a secure electronic case report form (eCRF).

Inclusion

Verification of inclusion and non-inclusion criteria, clinical examination, provision of study information, and collection of non-opposition consent.

Follow-up and Duration of Patient Participation

Inclusion period : 2 years
Patient participation duration: 1 year
Overall study duration: 3 years

The management of patients included in this study will not differ from standard clinical care routinely recommended for this condition.

5. STUDY POPULATION

5.1. Description of the Source Population

Patients with advanced-stage, unresectable, non-irradiable, or metastatic non-small cell lung cancer (NSCLC) discussed at the multidisciplinary thoracic oncology meetings (MDT) of the University Hospital of Brest and the Hospital of Morlaix, for whom first-line chemo-immunotherapy has been recommended.

5.2 Inclusion and Non-inclusion Criteria

Inclusion criteria

- Adult patients aged ≥ 18 years.
- Histologically confirmed advanced, unresectable, non-irradiable, or metastatic NSCLC.
- Treatment-naïve.
- Eligible for first-line anti–PD-1-based chemo-immunotherapy.
- Having provided non-opposition consent in accordance with French regulations for non-interventional research.
- Eligible for baseline 18F-FDG LAFOV PET/CT performed within 21 days before initiation of treatment

Non-Inclusion Criteria

- Presence of an actionable oncogenic driver mutation with available first-line targeted therapy (EGFR, ALK, ROS1, RET).
- Pregnancy or breastfeeding.
- Histology other than NSCLC.

- Not eligible for chemo-immunotherapy.
- Not eligible for 18F-FDG LAFOV PET/CT imaging.
- Refusal to participate.

5.3 Feasibility

Approximately 100 patients per year are discussed at the thoracic oncology MDT of the University Hospital of Brest and 50 patients per year at the Hospital of Morlaix with a diagnosis of advanced-stage NSCLC.

6. RECRUITMENT AND PARTICIPANT INFORMATION PROCEDURES

Recruitment will be conducted prospectively, following approval by the Ethics Committee (Comité de Protection des Personnes), by one of the designated investigators. Eligible patients will be identified during the initial thoracic oncology consultation. These will be patients diagnosed with advanced-stage non-small cell lung cancer (NSCLC) who are eligible for standard first-line chemo-immunotherapy.

Patients will be provided with complete, fair, and comprehensible information regarding the study objectives, their right to refuse participation, and their right to withdraw at any time without consequence. All this information will be presented in a written information and non-opposition form, which will be handed to the patient during the consultation.

The patient’s participation and non-opposition will be documented in their medical record.

7. STATISTICAL ANALYSIS

7.1. Statistical Responsibility and Data Storage

Statistical analyses will be performed by the biostatistics team of the Clinical Research and Innovation Department (Direction de la Recherche Clinique et de l’Innovation, DRCI) at the University Hospital of Brest.

7.2. Sample Size Justification

The 1-year progression-free survival (PFS) rates are estimated at 35.8% for squamous cell carcinoma and 38.8% for non-squamous carcinoma.

The imaging parameters to be evaluated include:

- Standard parameters: SUVmax, SUVpeak, MTV (metabolic tumour volume), and TLG (total lesion glycolysis).
- Textural parameters: entropy, homogeneity, HGZE (high grey-level zone emphasis), LGZE (low grey-level zone emphasis), LRE (long-run emphasis), and SRE (short-run emphasis).
- Dynamic parameters: Ki (net influx rate) and DV (distribution volume).

The objective is to determine optimal thresholds for each parameter using ROC curve analysis and to estimate sensitivity and specificity values for each parameter. Assuming that 120 patients are included, with approximately 60% experiencing disease progression at 1 year, the expected precision (half-width of the 95% confidence interval) would be approximately 10% for sensitivity and 12% for specificity, assuming observed values of around 80%.

7.3. Statistical Analysis Methods

Primary Endpoint

Progression-free survival (PFS), defined as the time from diagnosis to progression or death from any cause, whichever occurs first, will be used to evaluate the prognostic value of 18F-FDG LAFOV PET imaging. The primary endpoint is 1-year PFS.

PET parameters to be analysed:

Radiomic features:

First-order (standard) features:

SUVmax: maximum standardised uptake value within the tumour;

SUVpeak: highest possible mean value within a spherical VOI of 1 cm^3 located in the tumour;

MTV (metabolic tumour volume): total tumour volume in mL, including primary tumour and metastases, measured using different thresholding methods (40% SUVmax);

TLG (total lesion glycolysis): defined as MTV × SUVmean (automatically calculated as the mean SUV within each VOI).

Higher-order (textural) features: entropy, homogeneity, HGZE, LGZE, LRE, and SRE.

Dynamic features:
Derived from a linear regression model using the Patlak graphical approach:

Ki (net influx rate): tumoral uptake coefficient (mL/min/100 mL);

DV (distribution volume): expressed as a percentage.

A ROC (receiver operating characteristic) analysis will be performed to determine optimal cut-off values dividing patients into two prognostic groups (favourable vs unfavourable 1-year PFS). The area under the curve (AUC) will be calculated for each PET parameter, along with corresponding sensitivity, specificity, positive predictive value (PPV), and negative predictive value (NPV).

Progression-free survival curves will be estimated using the Kaplan–Meier method and compared between groups (based on the determined cut-off) using the log-rank test. Reported p-values will be considered exploratory due to the multiplicity of comparisons and the data-driven nature of cut-off selection.

Secondary Endpoints:

A1–A3: Correlations between quantitative 18F-FDG LAFOV PET parameters and histological, molecular, or biological markers will be estimated using Pearson's correlation coefficient with 95% confidence intervals. Associations between PET parameters and binary markers (presence/absence) will be assessed by comparing means using Student's t-test or Wilcoxon test as appropriate.

B: Agreement between reconstruction methods and the reference method (direct IDIF) will be assessed using Bland–Altman plots and intraclass correlation coefficients (ICC).

C: Agreement between quantitative parameters from 18F-FDG LAFOV PET acquisition and those obtained from post-acquisition degraded "SAFOV-like" PET reconstruction will also be evaluated using Bland–Altman plots and ICC.

D: Factors associated with overall survival (OS) will be explored using Cox proportional hazards models, and those associated with objective response using logistic regression models.

E: Safety and quality of life will be analysed descriptively, using frequency distributions, means, medians, and quartiles.

An interim descriptive analysis will be performed after inclusion of the first 40 patients. Given the exploratory nature of the study, no adjustment for multiple testing will be applied.

7.4 Handling of Missing, Unused, or Invalid Data

Analyses will be performed using available data only.

7.5 Analysis Population

All patients who undergo 18F-FDG LAFOV PET/CT and receive at least one cycle of chemo-immunotherapy will be included in the analysis.

8. DATA HANDLING AND STORAGE OF RESEARCH DOCUMENTS AND DATA

8.1. Case report form

8.2 Data Collected in the Case Report Form

Demographic data:

- First letter of last name, first letter of first name, inclusion number, age at diagnosis (in years), and sex.

Baseline clinical data:

- Medical history: chronic obstructive pulmonary disease (COPD), venous thromboembolism.
- Performance status, smoking status (non-smoker, current smoker, former smoker), occupational asbestos exposure, weight loss.

Baseline biological data:

- Neutrophil, lymphocyte, and eosinophil counts; albumin and lactate dehydrogenase (LDH) levels.

Histopathological data:

- Histological subtype, PD-L1 status, molecular profile (KRAS, BRAF, HER2, MET, NTRK, NRG1, KEAP1, NFE2L2, STK11, TP53, SMARCA4, and other relevant biomarkers).
- Cancer stage at diagnosis (TNM classification): locally advanced unresectable, non-irradiable, or metastatic disease.
- Metastatic sites.

Therapeutic data:

- Previous or concomitant local treatment: surgery or radiotherapy (dose and fractionation).
- Systemic treatment received:

- Type: platinum-doublet chemotherapy combined with anti–PD-1 therapy.
- Date of first treatment cycle.
- Date of last treatment cycle.
- Date and reason for treatment discontinuation.

- Adverse events related to systemic treatment, graded according to the CTCAE v5.0 classification.
- Concomitant medications: granulocyte colony-stimulating factor (G-CSF), corticosteroids (with dosage).
- Date of tumour progression.
- Subsequent systemic therapies administered after progression.

Imaging data:

- Date of baseline 18F-FDG LAFOV PET/CT.
- Date of first systemic response imaging.
- Date and type of best systemic response imaging (complete response, partial response, stable disease, or progression).
- Date of progression imaging.

PET/CT imaging parameters:

- Radiomic features:
  - *First-order (standard) parameters:*
    - SUVmax: maximum standardised uptake value within the tumour.
    - SUVpeak: highest possible mean SUV value within a spherical VOI of 1 cm^3 inside the tumour.
    - MTV (metabolic tumour volume): total tumour volume (in mL), including primary and metastatic lesions, measured using various thresholding methods (40% SUVmax).
    - TLG (total lesion glycolysis): expressed in grams (g), defined as MTV × SUVmean (automatically calculated as the mean SUV within each VOI).
  - *Higher-order (textural) features:* entropy, homogeneity, HGZE (high grey-level zone emphasis), LGZE (low grey-level zone emphasis), LRE (long-run emphasis), and SRE (short-run emphasis).
- Dynamic parameters:
  Derived from a linear regression model using the Patlak graphical approach:
  - Ki (net influx rate): tumoural uptake coefficient (mL/min/100 mL).
  - DV (distribution volume): expressed as a percentage.

Follow-up data:

- Date of last known contact.
- If deceased, date of death.

8.3 Confidentiality

Source documents, defined as any original record or material that allows verification of the existence or accuracy of data or facts recorded during the study, will be retained by the investigator or by the hospital if included in the institutional medical records.

Individuals with direct access to these documents will take all necessary precautions to ensure the confidentiality of information relating to the participants, particularly concerning their identity and the results obtained. These individuals, as well as the investigators themselves, are bound by professional confidentiality under Articles 226-13 and 226-14 of the French Penal Code.

During and after the study, the data collected on participants and transmitted by investigators will be coded. Under no circumstances shall these data include the participants' names or addresses in plain text.

Only the first letter of the last name and first letter of the first name will be recorded, along with a unique coded identification number corresponding to the order of inclusion in the study.

A paper correspondence table will be maintained at each study centre, containing the participant's code number, initials, full identity, date of birth, and date of inclusion. This correspondence list will be stored in the investigator's site file for five years after the end of the study.

8.4. CNIL Déclaration

This study falls under the framework of the French "Méthodologie de Référence" MR-003, pursuant to Article 54, paragraph 5 of Law No. 78-17 of 6 January 1978, as amended, relating to data protection and privacy (Informatique et Libertés). This framework was validated by Decision No. 2018-154 of 3 May 2018. The sponsor, University Hospital of Brest (CHU de Brest), has signed a formal commitment of compliance with this "Méthodologie de Référence"

8.5 Data Processing

Clinical data collection will rely on the implementation of an electronic clinical database derived from an electronic Case Report Form (eCRF).

Any detected inconsistencies will generate data queries for clarification and potential correction.

Data entry will be performed by Dr Margaux Geier and Dr Karim Amrane using a secure eCRF accessible via a personal login and password. Access to the database will be password-protected for enhanced data security. Prof. Ronan Abgral, co-investigator, will also have full access to all study data.

Data flow:

Dr Margaux Geier will personally visit the participating sites to centralise all collected data.

8.6 Data archiving

The electronic study data may be stored on the institution’s secure shared network for up to two years after the final publication of study results, or, in the absence of publication, until the final study summary report is signed.

In addition, the electronic data and all study-related documents will be archived for five years after study completion by the investigators.

Archiving will consist of securely storing a copy of all electronic data and paper documents in a locked cabinet within the institution.

9. ETHICAL AND LEGAL ASPECTS

9.1. Legal Obligations

9.1.1. Role of the sponsor

The University Hospital of Brest (CHU de Brest) is the sponsor of this study, in accordance with Article L.1121-1, paragraph 3 of the French Public Health Code (Code de la Santé Publique).

The sponsor is responsible for registering the study with the French National Agency for Medicines and Health Products Safety (ANSM) and submitting the application for review by a randomly selected Ethics Committee (Comité de Protection des Personnes, CPP).

A copy of the CPP opinion and the study summary will be transmitted to the competent authority.

Upon inclusion of the first participant, the sponsor will immediately notify the CPP of the actual study start date, defined as the date of signature of the consent form by the first participant.

The end of study date will be reported by the sponsor to the CPP within 90 days. The end of the study corresponds to the completion of participation by the last enrolled participant, or, where applicable, to the endpoint defined in the protocol.

9.1.2. Submission to the Ethics Committee

The CPP approval and opinion are referenced in the information and non-opposition form provided to study participants.

9.1.3. Notification of Associated Centres

The sponsor is responsible for obtaining agreement from all participating institutions and investigators involved in the study.

9.2. Protocol Amendments

Any substantial modification to the study protocol must be submitted by the sponsor to the Comité de Protection des Personnes (CPP) for approval, to ensure that the proposed changes do not compromise the protection, safety, or rights of the study participants.

9.3. Final Study Report

The final study summary report will be drafted by the coordinating principal investigator in collaboration with the study statistician. After study completion, this report will be transmitted to the sponsor as soon as possible.

10. PUBLICATION AND DATA OWNERSHIP

The University Hospital of Brest (CHU de Brest) is the legal owner of all study data. No data may be used, disclosed, or transmitted to third parties without prior written authorisation from the sponsor.
The name “CHU de Brest” must appear in the institutional address of all authors.
Scientific communications and reports resulting from this study will be prepared under the supervision of the coordinating principal investigator.
Co-authors of reports and publications will include all investigators and clinicians involved in the study, in proportion to their contribution, as well as the biostatistician and collaborating researchers.
Publication policy will follow international authorship and reporting guidelines (N Engl J Med, 1997; 336:309–315).
The study will be registered on a publicly accessible clinical trial website (ClinicalTrials.gov) prior to the inclusion of the first participant.

11. REFERENCES

1. Siegel RL, Miller KD, Jemal A. Cancer statistics, 2018. CA Cancer J Clin. 2018 Jan;68(1):7-30. doi: 10.3322/caac.21442.

2. Sung H, Ferlay J, Siegel RL, et al. Global Cancer Statistics 2020: GLOBOCAN Estimates of Incidence and Mortality Worldwide for 36 Cancers in 185 Countries. CA Cancer J Clin. 2021 May;71(3):209-249. doi: 10.3322/caac.21660.

3. Debieuvre D, Molinier O, Falchero L et al; Study Group KBP-2020-CPHG; KBP-2020-CPHG. Lung cancer trends and tumor characteristic changes over 20 years (2000-2020): Results of three French consecutive nationwide prospective cohorts' studies. Lancet Reg Health Eur. 2022 Aug 29;22:100492. doi: 10.1016/j.lanepe.2022.100492.

4. Brahmer J, Reckamp KL, Baas P, et al. Nivolumab versus Docetaxel in Advanced Squamous-Cell Non-Small-Cell Lung Cancer. N Engl J Med. 2015 Jul 9;373(2):123-35. doi: 10.1056/NEJMoa1504627.

5. Borghaei H, Paz-Ares L, Horn L, et al. Nivolumab versus Docetaxel in Advanced Nonsquamous Non-Small-Cell Lung Cancer. N Engl J Med. 2015 Oct 22;373(17):1627-39. doi: 10.1056/NEJMoa1507643.

6. Herbst RS, Baas P, Kim DW, et al. Pembrolizumab versus docetaxel for previously treated, PD-L1-positive, advanced non-small-cell lung cancer (KEYNOTE-010): a randomised controlled trial. Lancet. 2016 Apr 9;387(10027):1540-1550. doi: 10.1016/S0140-6736(15)01281-7.

7. Gandhi L, Rodríguez-Abreu D, Gadgeel S, et al; KEYNOTE-189 Investigators. Pembrolizumab plus Chemotherapy in Metastatic Non-Small-Cell Lung Cancer. N Engl J Med. 2018 May 31;378(22):2078-2092. doi: 10.1056/NEJMoa1801005.

8. Paz-Ares L, Luft A, Vicente D, et al; KEYNOTE-407 Investigators. Pembrolizumab plus Chemotherapy for Squamous Non-Small-Cell Lung Cancer. N Engl J Med. 2018 Nov 22;379(21):2040-2051. doi: 10.1056/NEJMoa1810865.

9. Garassino MC, Gadgeel S, Speranza G, et al. Pembrolizumab Plus Pemetrexed and Platinum in Nonsquamous Non-Small-Cell Lung Cancer: 5-Year Outcomes From the Phase 3 KEYNOTE-189 Study. J Clin Oncol. 2023 Apr 10;41(11):1992-1998. doi: 10.1200/JCO.22.01989.

10. Novello S, Kowalski DM, Luft A, et al. Pembrolizumab Plus Chemotherapy in Squamous Non-Small-Cell Lung Cancer: 5-Year Update of the Phase III KEYNOTE-407 Study. J Clin Oncol. 2023 Apr 10;41(11):1999-2006. doi: 10.1200/JCO.22.01990.

11. Dagogo-Jack I, Shaw AT. Tumour heterogeneity and resistance to cancer therapies. Nat Rev Clin Oncol. 2018 Feb;15(2):81-94. doi: 10.1038/nrclinonc.2017.166.

12. Borghaei H, Langer CJ, Paz-Ares L, et al. Pembrolizumab plus chemotherapy versus chemotherapy alone in patients with advanced non-small cell lung cancer without tumor PD-L1 expression: A pooled analysis of 3 randomized controlled trials. Cancer. 2020 Nov 15;126(22):4867-4877. doi: 10.1002/cncr.33142.

13. Salaün PY, Abgral R, Malard O, et al. Good clinical practice recommendations for the use of PET/CT in oncology. Eur J Nucl Med Mol Imaging. 2020 Jan;47(1):28-50. doi: 10.1007/s00259-019-04553-8.

14. Abgral R, Bourhis D, Salaun PY. Clinical perspectives for the use of total body PET/CT. Eur J Nucl Med Mol Imaging. 2021 Jun;48(6):1712-1718. doi: 10.1007/s00259-021-05293-4.

15. Hatt M, Tixier F, Pierce L, et al. Characterization of PET/CT images using texture analysis: the past, the present… any future? Eur J Nucl Med Mol Imaging. 2017 Jan;44(1):151-165. doi: 10.1007/s00259-016-3427-0.

16. Manafi-Farid R, Karamzade-Ziarati N, Vali R, et al. 2-[^{18}F]FDG PET/CT radiomics in lung cancer: An overview of the technical aspect and its emerging role in management of the disease. Methods. 2021 Apr;188:84-97. doi: 10.1016/j.ymeth.2020.05.023.

17. Reuzé S, Schernberg A, Orlhac F, et al. Radiomics in Nuclear Medicine Applied to Radiation Therapy: Methods, Pitfalls, and Challenges. Int J Radiat Oncol Biol Phys. 2018 Nov 15;102(4):1117-1142. doi: 10.1016/j.ijrobp.2018.05.022.

18. Zaidi H, Karakatsanis N. Towards enhanced PET quantification in clinical oncology. Br J Radiol. 2018 Jan;91(1081):20170508. doi: 10.1259/bjr.20170508.

19. Karakatsanis NA, Lodge MA, Tahari AK, Zhou Y, Wahl RL, Rahmim A. Dynamic whole-body PET parametric imaging: I. Concept, acquisition protocol optimization and clinical application. Phys Med Biol. 2013 Oct 21;58(20):7391-418. doi: 10.1088/0031-9155/58/20/7391.

20. Pavoine M, Thuillier P, Karakatsanis N et al. Clinical application of a population-based input function (PBIF) for a shortened dynamic whole-body FDG-PET/CT protocol in patients with metastatic melanoma treated by immunotherapy. EJNMMI Phys. 2023 Dec 8;10(1):79. doi: 10.1186/s40658-023-00601-3.

21. Thuillier P, Bourhis D, Metges JP, et al. Prospective study of dynamic whole-body 68Ga-DOTATOC-PET/CT acquisition in patients with well-differentiated neuroendocrine tumors. Sci Rep. 2021 Mar 1;11(1):4727. doi: 10.1038/s41598-021-83965-9.

22. Sari, H., Teimoorisichani, M., Mingels, C. et al. Quantitative evaluation of a deep learning-based framework to generate whole-body attenuation maps using LSO background radiation in long axial FOV PET scanners. Eur J Nucl Med Mol Imaging 49, 4490–4502 (2022). https://doi.org/10.1007/s00259-022-05909-3

23. Wang D, Qiu B, Liu Q, et al. Patlak-Ki derived from ultra-high sensitivity dynamic total body [18F]FDG PET/CT correlates with the response to induction immuno-chemotherapy in locally advanced non-small cell lung cancer patients. Eur J Nucl Med Mol Imaging. 2023 Sep;50(11):3400-3413. doi: 10.1007/s00259-023-06298-x.

24. Socinski MA, Nishio M, Jotte RM, et al. IMpower150 Final Overall Survival Analyses for Atezolizumab Plus Bevacizumab and Chemotherapy in First-Line Metastatic Nonsquamous NSCLC. J Thorac Oncol. 2021 Nov;16(11):1909-1924. doi: 10.1016/j.jtho.2021.07.009. Epub 2021 Jul 24. PMID: 34311108.

25. Eisenhauer EA, Therasse P, Bogaerts J, et al. New response evaluation criteria in solid tumours: revised RECIST guideline (version 1.1). Eur J Cancer. 2009 Jan;45(2):228–47.

26. Wahl RL, Jacene H, Kasamon Y et al. From RECIST to PERCIST: Evolving Considerations for PET response criteria in solid tumors. J Nucl Med. 2009 May;50 Suppl 1(Suppl 1):122S-50S. doi: 10.2967/jnumed.108.057307.

27. Common Terminology Criteria for Adverse Events (CTCAE). Available from : https://ctep.cancer.gov/protocolDevelopment/electronic_applications/docs/CTCAE_v5_QuickReference_8.5x11.pdf
