# Supplementary material for: PRognostic and predictive potential Of multiparametric dynamic whole-body 18F-FDG PET Imaging using a Long axial field-of-view (LAFOV) system for FIRST-line chemo-immunotherapy efficacy in advanced non-small cell lung cancer: PROFIL-1 study protocol
Source: PLoS One. 2026 Apr 3;21(4):e0345990. doi: 10.1371/journal.pone.0345990 (PMC13048445; doi:10.1371/journal.pone.0345990)
Supplement: S2 File — Original French version of the PROFIL-1 study protocol approved by the Comité de Protection des Personnes Sud-Est VI. (PDF) [file pone.0345990.s002.pdf]

**PROMOTEUR :**	CHU DE BREST
**PROTOCOLE DE RECHERCHE IMPLIQUANT LA PERSONNE HUMAINE DE CATEGORIE 3**	
**ACRONYME**<br><br>**CODE ESSAI**<br><br>**N° IDRCB**	**PROFIL-1**<br><br>29BRC24.0049<br><br>**2024-A00561-46**
**TITRE COMPLET**<br><br>**INDICATION(S) (CIBLE)**	**PR**edictive value **O**f multiparametric dynamic whole-body **F**DG-PET **I**maging on a **L**AFOV system for **first**-line chemo-immunotherapy efficacy in advanced non-small-cell lung cancer (NSCLC) (**PROFIL-1**)<br><br>Cancer Bronchique Non à Petites Cellules
**CO-INVESTIGATEURS COORDINATEURS**	**Dr Margaux GEIER**<br>Oncologie Médicale CHU Brest<br>02 30 33 80 30<br>**Pr Ronan ABGRAL**<br>Médecine Nucléaire CHU Brest
**N° DE VERSION DU PROTOCOLE**	V1.0
**DATE DU PROTOCOLE**	14/03/2024
**CPP**	Approuvé le 15 juillet 2024<br>Par le Comité de Protection des Personnes Sud Est VI
**CE DOCUMENT CONFIDENTIEL EST LA PROPRIETE DU CHU DE BREST AUCUNE INFORMATION NON PUBLIEE FIGURANT DANS CE DOCUMENT NE PEUT ETRE DIVULGUEE SANS AUTORISATION ECRITE PREALABLE DU CHU DE BREST**	

HISTORIQUE DES MODIFICATIONS

NUMERO DE VERSION (APRES MODIFICATION)	DATE	JUSTIFICATION DE LA MODIFICATION

SIGNATURES

SIGNATURE DU PROMOTEUR

NOM : CHU BREST

Signature : CHU Brest Date : 15/07/2024

SIGNATURE DE L'INVESTIGATEUR COORDINATEUR

J'ai lu l'ensemble des pages du protocole de la recherche dont le CHU de BREST est le promoteur. Je confirme qu'il contient toutes les informations nécessaires à la conduite de la recherche. Je m'engage à réaliser la recherche en respectant le protocole et les termes et conditions qui y sont définis. Je m'engage à réaliser la recherche en respectant :

- les principes de la "Déclaration d'Helsinki",
- les règles et recommandations de bonnes pratiques cliniques internationales (ICH-E6) et française (règles de bonnes pratiques cliniques pour les recherches impliquant la personne humaine)
- la législation nationale et la réglementation relative aux RIPH,

Je m'engage également à ce que les investigateurs et les autres membres qualifiés de mon équipe aient accès aux copies de ce protocole et des documents relatifs à la conduite de la recherche pour leur permettre de travailler dans le respect des dispositions figurant dans ces documents.

NOM: GEIER Margaux

Signature : M Geier Date : 15/07/2024

SIGNATURE DE L'INVESTIGATEUR PRINCIPAL DU CENTRE ASSOCIE

NOM : ________________________________ Centre : ______________

Signature : ………………………………………………… Date : ________________

SOMMAIRE

SIGNATURES 3
SIGNATURE DU PROMOTEUR 3
- Titre 5
- Promoteur 5
- Coordonnateurs 5
- Co-Investigateurs principaux 5
- Investigateurs associés 5
- Coordination et suivi de l'étude 6
- Scientifiques associés 6
- Méthodologiste – biostatisticien 6
2. JUSTIFICATION SCIENTFIQUE ET DESCRIPTION GENERALE DE LA RECHERCHE 11
2.1. Dénomination et description de la maladie 11
2.2. Contexte de la recherche et revue de la littérature 11
2.3 Résumé des résultats des études disponibles et pertinentes au regard de la recherche concernée 12
2.4. Justification de l'intérêt scientifique de l'étude 12
3. OBJECTIFS 12
3.1. Objectif principal 12
3.2. Objectifs secondaires 12
4. CONCEPTION ET DEROULEMENT DE LA RECHERCHE 13
4.1. Qualification de la recherche et des investigateurs. 13
4.2. Critères d'Evaluation 13
4.3. Déroulement de la recherche 14
Durée des inclusions : 2 ans 15
Durée de participation du patient : 1 an 15
Durée de l'étude : 3 ans 15
5. POPULATION ETUDIEE 15
5.1. Description de la population source 15
5.2 Critères d'inclusion et de non inclusion 15
5.3 Faisabilité 15
6. MODALITE DE RECRUTEMENT ET D'INFORMATION DES PERSONNES CONCERNEES 15
7. STATISTIQUES 16
7.1. Responsable de l'Analyse et lieu de conservation des données 16
7.2. Justification du nombre de sujets à inclure 16
7.3. Méthode d'analyse statistique 16
7.4 Méthode de prise en compte des données manquantes, inutilisées ou non valides 17
7.5 Choix des personnes prise à inclure dans l'analyse 17
8. TRAITEMENT DES DONNEES ET CONSERVATION DES DOCUMENTS ET DES DONNEES RELATIVES A LA RECHERCHE 17
8.1. Cahier d'observation 17
8.2. Identification des données recueillies dans le cahier d'observation 18
8.4. Déclaration CNIL 19
8.5. Traitement des données 20
8.6. Archivage des données 20
9. ASPECT ETHIQUES ET LEGAUX 20
9.1. Obligations légales. 20
9.2. Modification de la recherche 21
9.3. Résumé final de l'étude 21
10. PUBLICATION ET PROPRIETE DES DONNEES 21
11. BIBLIOGRAPHIE 22

- **Titre:**

  PRedictive value **O**f multiparametric dynamic whole-body **F**DG-PET **I**maging on a **L**AFOV system for **first**-line chemo-immunotherapy efficacy in advanced non-small-cell lung cancer (NSCLC) (**PROFIL-1**)

  Valeur prédictive d'une imagerie dynamique multiparamétrique TEP-FDG corps entier sur système LAFOV de l'efficacité d'une chimio-immunothérapie de 1ère ligne dans le cancer bronchique non à petites cellules (CBNPC) de stade avancé (**PROFIL-1**)

- ***Promoteur :***

  CHU de Brest
  2 avenue Foch-29609 Brest cedex

- ***Coordonnateurs***

  - Dr Margaux GEIER – Onco-pneumologue
    Service d'Oncologie Médicale, CHU MORVAN, 2 avenue FOCH, 29 200 Brest
    margaux.geier@chu-brest.fr
    02 0 33 80 30

- ***Co-Investigateurs principaux***

  - Pr Ronan ABGRAL
    Service de Médecine Nucléaire CHU Brest, 2 avenue FOCH, 29 200 Brest
    ronan.abgral@chu-brest.fr
    02 98 22 30 69
  - Dr Karim AMRANE (Oncologie CH Morlaix)
    KAmrane@ch-morlaix.fr
    02 98 62 60 38

- ***Investigateurs associés***

  - Dr François LUCIA (Radiothérapie CHU Brest)
    francois.lucia@chu-brest.fr
    02 98 23 81 57

  - Dr Vincent BOURBONNE (Radiothérapie CHU Brest)
    vincent.bourbonne@chu-brest.fr
    02 29 02 02 89

  - Pr Pierre-Yves LE ROUX (Médecine Nucléaire CHU Brest)
    pierre-yves.leroux@chu-brest.fr
    02 98 22 33 27

  - Pr Pierre-Yves SALAUN (Médecine Nucléaire CHU Brest)
    pierre-yves.salaun@chu-brest.fr
    02 98 22 33 27

- David BOURHIS (Radiophysicien, Médecine Nucléaire CHU Brest)
  David.bourhis@chu-brest.fr
  02 98 22 30 50

- Dr Renaud DESCOURT (Oncologie Médicale, CHU Brest)
  renaud.descourt@chu-brest.fr
  02 98 22 37 40

- Dr Gilles QUERE (Oncologie Médicale, CHU Brest)
  gilles.quere@chu-brest.fr
  02 98 22 37 40

- Dr Jessica NGUYEN (Oncologie Médicale, CHU Brest)
  jessica.nguyen@chu-brest.fr
  02 98 22 37 40

- ***Coordination et suivi de l'étude***

Direction de la Recherche Clinique et de l'Innovation (DRCI)
Hôpital Morvan – 2 avenue Foch
29 609 Brest cedex

- ***Scientifiques associés***

- Pr COUTURAUD Francis (Pneumologie, CHU Brest)
  francis.couturaud@chu-brest.fr
  02 98 34 78 26

- Dr Nicolas KARAKATSANIS (Assistant Professor of Biomedical Engineering in Radiology, Weill Cornell Medicine, Cornell University, New York)
  nak2032@med.cornell.edu

- Dr Florent BESSON (Département de Médecine Nucléaire CH Bicêtre, Université Paris Saclay, UMR Inserm BIOMAPS, France)

- ***Méthodologiste – biostatisticien***

Unité bio statistique – DRCI de Brest

1. RESUME

TITRE ANGLAIS	**PR**edictive value **O**f multiparametric dynamic whole-body **F**DG-PET **I**maging on a **L**AFOV system for **first**-line chemo-immunotherapy efficacy in advanced non-small-cell lung cancer (NSCLC) (**PROFIL-1**)
TITRE FRANCAIS	Valeur prédictive d’une imagerie dynamique multiparamétrique TEP-FDG corps entier sur système LAFOV de l’efficacité d’une chimio-immunothérapie de 1ère ligne dans le cancer bronchique non à petites cellules (CBNPC) de stade avancé (**PROFIL-1**)
PROMOTEUR	CHU de Brest
INVESTIGATEUR PRINCIPAL OU COORDONATEUR	Dr Margaux GEIER (Oncologie CHU Brest)
ACRONYME	**PROFIL-1**
JUSTIFICATION DE L’ETUDE	Le cancer bronchique est la 1ère cause de mortalité par cancer en France et dans le monde, avec une survie relative à 5 ans d’environ 20 % tous stades confondus. Le diagnostic de la maladie est porté dans environ 70% des cas à un stade métastatique avancé non accessible à un traitement curatif. Les progrès de l'immunothérapie, et notamment des inhibiteurs de checkpoints de l’immunité (ICI), ont offert une opportunité d'améliorer le pronostic des patients atteints de cancers bronchiques non à petites cellules (CBNPC). La combinaison chimio-immunothérapie a plus récemment été validée pour le traitement du CBNPC en 1ère ligne métastatique.<br>Le CBNPC présente une forte hétérogénéité inter- et intra-tumorale, expliquant possiblement les différences de taux et durée de réponse au traitement. La TEP-TDM au FDG est une imagerie fonctionnelle actuellement indiquée pour l’évaluation thérapeutique des CBNPC métastatiques sous traitement systémique. La TEP grand-champ (TEP-GC) ou « LAFOV PET » est un système innovant de toute dernière génération et permettrait de mieux caractériser cette hétérogénéité tumorale. Elle présente deux avantages majeurs par rapport aux autres systèmes du marché (SAFOV) à savoir un gain en sensibilité de détection et une possibilité d'analyse dynamique corps entier multi-organes. Ainsi la recherche de biomarqueurs d’imagerie en TEP-TDM pour prédire précocement la réponse au traitement est un enjeu majeur en oncologie pour tendre vers une médecine personnalisée.<br>Notre hypothèse est que la TEP-FDG LAFOV permettrait d’améliorer la prédiction d’efficacité d’une chimio-immunothérapie de 1ère ligne chez des patients atteints d’un CBNPC de stade avancé en complément des biomarqueurs usuels.
POPULATION CONCERNEE	Patients présentant un CBNPC de stade avancé chez qui l’indication d’un traitement par chimio-immunothérapie est retenue en 1ère ligne.
OBJECTIF PRINCIPAL	Etudier les performances pronostiques d’une analyse multiparamétrique (radiomique et dynamique) corps entier en

	LAFOV-PET sur l'efficacité en terme de survie sans progression (SSP) d'une cohorte de patients atteints d'un CBNPC de stade avancé traités par chimio-immunothérapie en 1ère ligne.
OBJECTIFS SECONDAIRES	A1- Evaluer l'association entre les paramètres quantitatifs en TEP-FDG LAFOV et les marqueurs histologiques des tumeurs (histologie épidermoïde/non-épidermoïde, statut PD-L1 (négatif, 1-49%, ≥50%), Ki67, VEGF, TIL + autres marqueurs d'intérêt).<br><br>A2- Rechercher une corrélation entre les paramètres quantitatifs en TEP-FDG LAFOV et les marqueurs de biologie moléculaire des tumeurs (KRAS, BRAF, HER2, MET, NTRK, NRG1, KEAP1, NFE2L2, STK11, TP53, SMARCA4 + autres marqueurs d'intérêt).<br><br>A3- Rechercher une corrélation entre les paramètres quantitatifs en TEP-FDG LAFOV et les paramètres biologiques du patient (taux polynucléaires neutrophiles, lymphocytes, LIPI score, taux d'éosinophiles, taux de CRP, taux LDH, taux d'albumine).<br><br>B- Comparer des méthodes de reconstructions Patlak direct et indirect avec PBIF ou IDIF en TEP-FDG LAFOV pour le calcul des paramètres dynamiques Ki et DV.<br><br>C- Comparer les paramètres quantitatifs issus de l'acquisition TEP-FDG LAFOV avec ceux issus d'une reconstruction post-acquisition dégradée TEP SAFOV « like ».<br><br>D- Evaluer d'autres critères d'efficacité de la combinaison chimio-immunothérapie (survie globale, taux de réponse objective).<br><br>E- Etudier la corrélation entre les paramètres quantitatifs en TEP-FDG LAFOV et la survenue d'une toxicité.
CRITERE D'EVALUATION PRINCIPAL	Taux de survie sans progression à 1 an
CRITERES D'EVALUATION SECONDAIRES	A1- Paramètres d'imagerie quantitatifs en TEP-FDG LAFOV et les marqueurs histologiques des tumeurs (histologie (épidermoïde vs non-épidermoïde, statut PD-L1 (négatif, 1-49%, ≥50%), Ki67, VEGF, TIL + autres marqueurs d'intérêt)<br><br>A2- Paramètres d'imagerie quantitatifs en TEP-FDG LAFOV et les marqueurs de biologie moléculaire des tumeurs (KRAS, BRAF, HER2, MET, NTRK, NRG1, KEAP1, NFE2L2, STK11, TP53, SMARCA4 + autres marqueurs d'intérêt).<br><br>A3- Paramètres d'imagerie quantitatifs en TEP-FDG LAFOV et les paramètres biologiques (taux polynucléaires neutrophiles, lymphocytes, LIPI score, taux d'éosinophiles, taux de CRP, taux LDH, taux d'albumine).<br><br>B- Paramètres dynamiques Ki et DV issus des reconstructions Patlak direct et indirect avec IDIF ou PBIF.<br><br>C- Nombre de lésions tumorales et rapport signal/bruit

	D- Etudier la valeur prédictive de la survie globale, définie comme le temps entre le début du traitement et la survenue du décès quelle qu'en soit la cause, et du taux de réponse objective systémique selon les critères RECIST et/ou PERCIST.<br><br>E- Tolérance (effets indésirables selon la classification CTCAE v5.0[27]; scores de qualité de vie EQ-5D-5L et EORTC QLQ-C30 évalués au baseline, à 3 mois, 6 mois et 1 an).
METHODOLOGIE	Etude pilote non interventionnelle prospective multicentrique (CHU Brest + CH Morlaix)
STATISTIQUE	L'objectif serait d'établir des seuils pertinents pour chaque paramètre (à l'aide d'une courbe ROC) et de calculer les valeurs de sensibilité/spécificité obtenues pour chaque paramètre. Avec 120 patients dont 60 % présenteront une progression tumorale à 1 an, il serait possible d'estimer les valeurs de sensibilité/spécificité avec une précision (1/2 amplitude de l'intervalle de confiance à 95 %) d'environ 10 % pour la sensibilité et 12 % pour la spécificité, en supposant des valeurs observées d'environ 80 %.
CRITERE D'INCLUSION	- Patient majeur ≥ 18 ans<br>- Présentant un CBNPC de stade avancé, non opérable, non irradiable ou métastatique<br>- Naïfs de traitement<br>- Eligible à une chimio-immunothérapie (anti-PD-1) en 1ère ligne<br>- Ayant formulé sa non-opposition<br>- Eligible à l'examen TEP-FDG LAFOV (délai de moins de 21 jours avant l'initiation du traitement)
CRITERES DE NON-INCLUSION	- Patient mineur <18 ans<br>- Addiction oncogénique ciblable en 1ère ligne : *EGFR, ALK, ROS1, RET*<br>- Grossesse ou allaitement<br>- Autre histologie que CBNPC<br>- Non éligibilité à une chimio-immunothérapie<br>- Non éligibilité à l'examen TEP LAFOV<br>- Refus de participation
NOMBRE DE PATIENTS	L'estimation finale de la taille de l'échantillon est de 120 patients.
CALENDRIER	Durée des inclusions : 2 ans<br>Durée de participation du patient : 1 an<br>Durée de l'étude : 3 ans

RETOMBEES ATTENDUES	Les CBNPC sont des tumeurs de mauvais pronostic, notamment parce que la majorité des lésions sont métastatiques au diagnostic. Malgré l'existence des divers schémas thérapeutiques et les progrès récents depuis l'avènement de l'immunothérapie, cette affection reste un problème majeur de santé publique, en raison notamment d'une hétérogénéité de réponse intra- et inter-tumorale.<br><br>L'identification précoce des patients réfractaires au traitement serait un avantage indéniable pour leur survie mais aussi pour l'économie de santé.<br><br>Malgré une évaluation attentive des facteurs pronostiques connus, il reste difficile de prédire de façon fiable la survie des patients. L'identification de nouveaux paramètres pré-thérapeutiques en TEP/TDM tels que la diffusion spatio-temporelle du traceur (combinant approche radiomique et cinétique) pourrait être d'un grand intérêt en termes de stratégie thérapeutique. Le système TEP LAFOV, dont peu de machines sont implantées actuellement dans le monde (1ère en France métropolitaine), semble pouvoir répondre à cet objectif. Ainsi, un sous-groupe de patients à mauvais pronostique pourrait ainsi bénéficier d'une escalade thérapeutique soit par intensification du traitement systémique avec ajout d'autres agents soit par traitement stéréotaxique de lésions réfractaires oligométastatiques.

2. JUSTIFICATION SCIENTFIQUE ET DESCRIPTION GENERALE DE LA RECHERCHE

2.1. Dénomination et description de la maladie

Le cancer bronchique représente la 1ère cause de décès par cancer chez l'homme avec une survie relative à 5 ans d'environ 20 % tous stades confondus[1]. Le diagnostic de la maladie est porté dans près de 70% des cas à un stade avancé non accessible à un traitement curatif [2,3].

Les progrès de l'immunothérapie et notamment des inhibiteurs de checkpoints immunitaires (ICI) ont offert une opportunité d'améliorer les résultats pour les patients atteints de cancers bronchiques non à petites cellules (CBNPC) avec un taux de réponse avoisinant les 20 % [4,5,6]. Plus récemment, la combinaison chimio-immunothérapie a été validée pour le traitement du CBNPC en 1ère ligne métastatique[7,8]. Cependant, malgré une amélioration des durées de réponse et des taux de survie à long terme[9,10], certains patients ne tirent pas de bénéfice de ces nouvelles thérapies anti-cancéreuses, sans que l'on puisse le prédire de façon fiable. En effet, les CBNPC présentent une forte hétérogénéité inter- et intra-tumorale (clones cellulaires aux taux de prolifération variables et zones d'angiogenèse, d'hypoxie, de nécrose ou de fibrose différents...), expliquant possiblement les différences de réponse au traitement[11]. La caractérisation de cette hétérogénéité tumorale est un enjeu majeur en oncologie pour améliorer la prise en charge thérapeutique et tendre vers une médecine personnalisée adaptée à chaque patient[12].

2.2. Contexte de la recherche et revue de la littérature

La TEP-TDM au FDG est une imagerie fonctionnelle caractérisant le métabolisme glucidique des tumeurs et actuellement indiquée pour l'évaluation thérapeutique des cancers bronchiques métastatiques sous traitement systémique[13]. La possibilité de trouver des biomarqueurs de substitution en imagerie TEP-TDM (« surrogate markers ») pour mieux caractériser l'hétérogénéité tumorale et prédire précocement la survie et la réponse au traitement reste un enjeu crucial.

La TEP grand-champ (TEP-GC) ou « LAFOV PET » est un système innovant de toute dernière génération (moins de 15 machines en Europe, 1 en France) qui présente deux avantages majeurs par rapport aux autres systèmes du marché (SAFOV) à savoir un gain en sensibilité de détection et une possibilité d'analyse dynamique corps entier multi-organes[14]. Ces caractéristiques ouvrent des réelles voies d'optimisation de l'analyse multiparamétrique des images TEP.

L'analyse texturale (radiomique) en imagerie TEP, correspondant à une analyse de la distribution spatiale des voxels, permet le calcul de nombreux indices reflétant l'hétérogénéité tumorale[15]. Quelques études ont ainsi permis de montrer l'intérêt pronostique de l'analyse de texture pour le CBNPC en TEP au FDG[16]. Néanmoins, la qualité de cette analyse texturale est dépendante du bruit de l'image (donc de la performance intrinsèque du système) et reste limitée pour les petites lésions ou en mouvement, comme dans le poumon[17].

L'analyse dynamique 4D (dynTEP) a été proposée pour extraire des paramètres quantitatifs issus de l'analyse temporelle de la distribution du radiotraceur dans les voxels[18,19]. Elle permet d'estimer des paramètres cinétiques en utilisant notamment une modélisation basée sur une analyse Patlak-like, après une estimation de la fonction d'entrée plasmatique à partir des images (Image based input function IDIF) ou d'une population témoin (Population based input function PBIF)[20]. Les premières études sur les acquisitions dynTEP ont montré l'absence de corrélation linéaire entre les valeurs de SUV et de Ki[21], suggérant l'apport d'informations quantitatives supplémentaires à partir des données cinétiques et ouvrant de nouvelles perspectives pour l'évaluation pronostique de ces CBNPC[22].

La TEP-LAFOV pourrait résoudre ces limites actuelles avec des possibilité de reconstruction d'images avec une matrice haute résolution limitée en bruit et avec un excellent échantillonnage temporel (corps entier en 10-20 sec) pour l'optimisation de l'analyse radiomique et la quantification dynamique 4D.

2.3 Résumé des résultats des études disponibles et pertinentes au regard de la recherche concernée

Une seule étude a récemment montré l'intérêt potentiel de cette technique d'analyse multiparamétrique en TEP-LAFOV pour prédire la réponse au traitement par chimio-immunothérapie des CBNPC. Cependant, il s'agissait uniquement d'une analyse tumorale T sur des formes localement avancées de cancer en situation de traitement systémique d'induction[23].

2.4. Justification de l'intérêt scientifique de l'étude

Les CBNPC sont des tumeurs de mauvais pronostic, en lien avec la fréquence des maladies d'emblée métastatique au diagnostic. Malgré l'existence des divers schémas thérapeutiques et les progrès récents depuis l'avènement de l'immunothérapie, cette affection reste un problème majeur de santé publique, en raison notamment d'une hétérogénéité de réponse intra- et inter-tumorale.

L'identification précoce des patients réfractaires au traitement serait un avantage indéniable pour leur survie mais aussi pour l'économie de santé.

Malgré une évaluation attentive des facteurs pronostiques connus, il reste difficile de prédire de façon fiable la survie des patients. L'identification de nouveaux paramètres pré-thérapeutiques en TEP/TDM tels que la diffusion spatio-temporelle du traceur (combinant approche radiomique et cinétique) pourrait être d'un grand intérêt en termes de stratégie thérapeutique. Le système TEP-LAFOV dont peu de machines sont implantées actuellement dans le monde semble pouvoir répondre à cet objectif. Ainsi, un sous-groupe de patients à mauvais pronostique pourrait ainsi bénéficier d'une escalade thérapeutique soit par intensification du traitement systémique avec ajout d'autres agents[24] soit par traitement stéréotaxique de lésions réfractaires oligométastatiques.

3. OBJECTIFS

3.1. Objectif principal

Etudier les performances pronostiques d'une analyse multiparamétrique (radiomique et dynamique) corps entier en TEP-FDG LAFOV sur l'efficacité en terme de survie sans progression d'une cohorte de patients atteints d'un CBNPC de stade avancé traités par chimio-immunothérapie en 1ère ligne.

3.2. Objectifs secondaires

A1- Evaluer l'association entre les paramètres quantitatifs en TEP-FDG LAFOV et les marqueurs histologiques des tumeurs (histologie épidermoïde/non-épidermoïde, statut PD-L1 (négatif, 1-49%, ≥50%), Ki67, VEGF, TIL + autres marqueurs d'intérêt).

A2- Rechercher une corrélation entre les paramètres quantitatifs en TEP-FDG LAFOV et les marqueurs de biologie moléculaire des tumeurs (KRAS, BRAF, HER2, MET, NTRK, NRG1, KEAP1, NFE2L2, STK11, TP53, SMARCA4 + autres marqueurs d'intérêt).

A3- Rechercher une corrélation entre les paramètres quantitatifs en TEP-FDG LAFOV et les paramètres biologiques du patient (taux polynucléaires neutrophiles, lymphocytes, LIPI score, taux d'éosinophiles, taux de CRP, taux LDH, taux d'albumine).

B- Comparer les méthodes de reconstruction Patlak direct (Siemens) et indirect (PETkinetic) avec PBIF ou IDIF en TEP-FDG LAFOV pour le calcul des paramètres dynamiques Ki et DV.

C- Comparer les paramètres quantitatifs issus de l'acquisition TEP-FDG LAFOV avec ceux issus d'une reconstruction post-acquisition dégradée TEP SAFOV « like »

D- Evaluer d’autres critères d’efficacité de la combinaison chimio-immunothérapie (survie globale, taux de réponse objective)

E- Etudier la corrélation entre les paramètres quantitatifs en TEP-FDG LAFOV et la survenue d’une toxicité

4. CONCEPTION ET DEROULEMENT DE LA RECHERCHE

4.1. Qualification de la recherche et des investigateurs.

Etude pilote non interventionnelle prospective multicentrique pratiquée sur l’être humain en vue du développement des connaissances biologiques et médicales, dans laquelle les actes sont pratiqués et les produits utilisés de manière habituelle, sans procédure supplémentaire ou inhabituelle de diagnostic, de traitement ou de surveillance.

La prise en charge des patients atteints d’un CBNPC fait partie des domaines d’expertise des oncologues thoraciques investigateurs.
L’imagerie nucléaire est le domaine d’expertise des investigateurs travaillant au sein du service de médecine nucléaire.

4.2. Critères d’Evaluation

4.2.1. Critère principal d’évaluation

Survie sans progression (SSP), définie comme le temps entre la date de début du traitement et la date de progression ou de décès quelle qu’en soit la cause.

La SSP à 1 an sera choisie comme critère pour évaluer les performances pronostiques de la TEP-FDG LAFOV.

La progression tumorale sera évaluée selon les critères RECIST[25] sur les différents scanners de suivi et/ou selon les critères PERCIST[26] en cas de réalisation de TEP-FDG.

4.2.2. Critères d’évaluation secondaire

A1- Paramètres d’imagerie quantitatifs en TEP-FDG LAFOV et les marqueurs histologiques des tumeurs (histologie (épidermoïde vs non-épidermoïde, statut PD-L1 (négatif, 1-49%, ≥50%), Ki67, VEGF, TIL + autres marqueurs d’intérêt)

A2- Paramètres d’imagerie quantitatifs en TEP-FDG LAFOV et les marqueurs de biologie moléculaire des tumeurs (KRAS, BRAF, HER2, MET, NTRK, NRG1, KEAP1, NFE2L2, STK11, TP53, SMARCA4 + autres marqueurs d’intérêt).

A3- Paramètres d’imagerie quantitatifs en TEP-FDG LAFOV et les paramètres biologiques (taux polynucléaires neutrophiles, lymphocytes, LIPI score, taux d’éosinophiles, taux de CRP, taux LDH, taux d’albumine).

B- Paramètres dynamiques Ki et DV issus des reconstructions Patlak direct et indirect avec IDIF ou PBIF.

C- Nombre de lésions tumorales et rapport signal/bruit

D- Etudier la valeur prédictive de la survie globale, définie comme le temps entre le début du traitement et la survenue du décès quelle qu’en soit la cause, et du taux de réponse objective systémique selon les critères RECIST et/ou PERCIST.

E- Tolérance (effets indésirables selon la classification CTCAE v5.0[27]; scores de qualité de vie EQ-5D-5L et EORTC QLQ-C30 évalués au baseline, à 3 mois, 6 mois et 1 an).

4.3. Déroulement de la recherche

Description synthétique du schéma de l'étude (maximum 750 signes)

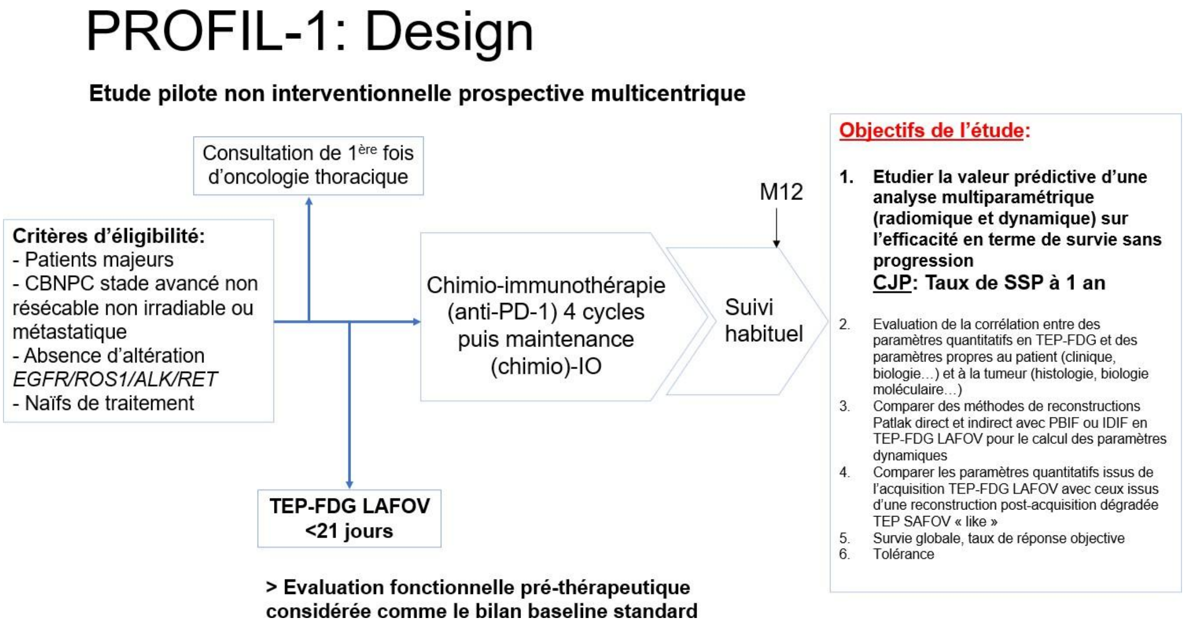

Modalité de recrutement

Le recrutement sera réalisé de manière prospective, dès accord du Comité de Protection des Personnes, par un des investigateurs déclarés. Les patients éligibles seront recrutés lors de la consultation de 1ère fois d'oncologie thoracique aux CHU de Brest et CH de Morlaix : patients atteints d'un CBNPC de stade avancé et éligibles à un traitement standard par chimio-immunothérapie en 1ère ligne.
- Les critères d'inclusion et de non-inclusion seront alors vérifiés puis la non–opposition du patient sera demandée lors de cette consultation via la remise d'un formulaire. Celui-ci contiendra une information complète, loyale, exprimée en des termes compréhensibles. Les objectifs de l'étude, leurs droits de refuser de participer à l'étude ou de la possibilité de se rétracter à tout moment y seront exposés. En cas de réponse positive lors de l'entretien, le patient pourra être inclus. En cas de réponse négative ou en l'absence de réponse, le patient ne sera pas inclus.
- Réalisation d'une TEP-FDG LAFOV baseline dans les 21 jours précédant le début du traitement par chimio-immunothérapie. Cet examen d'imagerie sera considéré comme le bilan d'imagerie habituel pré-thérapeutique.
- Suivi classique en oncologie ensuite.

Le recueil prospectif des données concernant les caractéristiques cliniques et biologiques des patients, les caractéristiques du cancer, mais aussi les données d'imagerie, pourra alors débuter et être intégré dans un CRF électronique sécurisé.

Inclusion

Vérification des critères d'inclusion et de non inclusion, examen clinique, information et recueil de la non-opposition.

Suivi et durée de participation du patient

Durée des inclusions : 2 ans
Durée de participation du patient : 1 an
Durée de l'étude : 3 ans

La prise en charge des patients inclus dans cette étude n'est pas modifiée par rapport à la prise en charge habituellement préconisée.

5. POPULATION ETUDIEE

5.1. Description de la population source

Patients présentant un CBNPC de stade avancé, non opérable, non irradiable ou métastatique, dont les dossiers sont présentés en RCP d'oncologie thoracique au CHU de Brest et au CH de Morlaix, chez qui l'indication d'un traitement par chimio-immunothérapie est retenue en 1ère ligne

5.2 Critères d'inclusion et de non inclusion

Critère d'inclusion

- Patients majeurs ≥ 18 ans
- Présentant un CBNPC de stade avancé, non opérable, non irradiable ou métastatique
- Naïfs de traitement
- Eligible à une chimio-immunothérapie (anti-PD-1) en 1ère ligne
- Ayant formulé sa non-opposition
- Eligible à l'examen TEP-FDG LAFOV dans un délai de moins de 21 jours avant l'initiation du traitement

Critère de Non-inclusion

- Patient mineur <18 ans
- Addiction oncogénique ciblable en 1ère ligne : *EGFR, ALK, ROS1, RET*
- Grossesse ou allaitement
- Autre histologie que CBNPC
- Non éligibilité à une chimio-immunothérapie
- Non éligibilité à l'examen TEP-FDG LAFOV
- Refus de participation

5.3 Faisabilité

Environ 100 patients/an sont présentés en RCP d'oncologie thoracique au CHU de Brest et 50 patients/an au CH de Morlaix avec un diagnostic de CBNPC de stade avancé.

6. MODALITE DE RECRUTEMENT ET D'INFORMATION DES PERSONNES CONCERNEES

Le recrutement sera réalisé de manière prospective, dès accord du Comité d'Ethique, par un des investigateurs déclarés. Les patients éligibles seront recrutés lors de la consultation de 1ère fois

d’oncologie thoracique : patients atteints d’un CBNPC de stade avancé et éligibles à un traitement standard par chimio-immunothérapie en 1ère ligne.
Les patients seront informés de façon complète et loyale, en des termes compréhensibles, des objectifs de l'étude, de leurs droits de refuser de participer à l'étude ou de la possibilité de se rétracter à tout moment. Toutes ces informations figureront sur un formulaire d’information et de non opposition remis au patient.
La participation du patient sera notée dans son dossier médical.

7. STATISTIQUES

7.1. Responsable de l’Analyse et lieu de conservation des données

Les analyses statistiques seront réalisées par l’équipe statistique de la DRCI du CHU de Brest.

7.2. Justification du nombre de sujets à inclure

Les taux de survie sans progression à 1 an sont estimés à 35,8% pour les carcinomes épidermoïdes et à 38,8% pour les carcinomes non-épidermoïdes.

Les différents paramètres d’imagerie sont les suivants :

- Standards: SUV max, SUV peak, MTV (metabolic tumour volume), TLG (total lesion glycolysis)
- Paramètres de texture: entropie, homogénéité, HGZE, LGZE, LRE, SRE
- Dynamiques: Ki (net influx rate), DV (volume de distribution)

L'objectif est d'établir des seuils pertinents pour chaque paramètre (à l'aide d'une courbe ROC) et de calculer les valeurs de sensibilité/spécificité obtenues pour chaque paramètre. Avec 120 patients dont environ 60 % présenteront une progression tumorale à 1 an, il serait possible d'estimer les valeurs de sensibilité/spécificité avec une précision (1/2 amplitude de l'intervalle de confiance à 95 %) d'environ 10 % pour la sensibilité et 12 % pour la spécificité, en supposant des valeurs observées d'environ 80 %.

7.3. Méthode d’analyse statistique

Critère de jugement principal :

La survie sans progression (SSP), définie comme la durée entre la date de diagnostic et la date de progression ou de décès, sera choisie comme critère d'évaluation de la valeur pronostique de la TEP-FDG. Le critère principal est la survie sans progression à 1 an.

Différents paramètres TEP seront analysés dans les études TEP :

- **Radiomique**
  - ***Standard (1er ordre)***
    - SUV max = valeur maximale du SUV sur la tumeur ;
    - SUV peak = valeur moyenne la plus élevée possible d'un VOI sphérique de 1 cm3 situé dans la tumeur ;
    - MTV (metabolic tumor volume) = volume total en mL incluant la tumeur primitive ou les métastases, mesuré à l'aide de différentes méthodes du seuil de 40 % SUV max ;
    - TLG (total lesion glycolysis) en grammes (g) défini comme MTV x SUV mean (automatiquement calculé comme le SUV moyen dans chaque VOI) ;

  - ***Textural features (2e et 3e ordres)***
    - Entropie, homogénéité, HGZE, LGZE, LRE et SRE.

- **Dynamique**

A partir d’un modèle de régression linéaire du graphique Patlak :

- Ki (net influx rate) = coefficient d'absorption tumorale en ml/min/100ml;
- DV (distribution volume) = volume de distribution en %

Une analyse ROC (Receiver-operating characteristic) sera effectuée pour déterminer la valeur seuil (cut-off) optimale qui divise les patients en 2 sous-groupes de mauvais ou de bon pronostic en termes de SSP pour chaque paramètre TEP. L'aire sous la courbe (AUC) sera calculée pour chacun des paramètres de la TEP, ainsi que les sensibilités, spécificités, valeurs prédictives positives et négatives pour les cut-offs choisis.

Les courbes de survie sans progression seront représentées à l'aide de la méthode de Kaplan-Meier et comparées (après dichotomie de l'échantillon selon le cut-off choisi) à l'aide d'un test du logrank (p-values données à titre exploratoire en raison de la multiplicité des comparaisons et du choix d'un cut-off guidé par les données).

Critères secondaires :

A1-A3 : Les corrélations entre les valeurs des paramètres quantitatifs en TEP-FDG LAFOV et celles des marqueurs histologiques, moléculaires ou biologiques seront estimées à l'aide d'un coefficient de corrélation de Pearson avec intervalle de confiance à 95%

L'association entre les paramètres quantitatifs en TEP-FDG et des marqueurs binaires (présence/absence) sera évaluée en comparant les moyennes entre les deux groupes (présence/absence du marqueur) à l'aide d'un test de Student ou de Wilcoxon si requis.

B – Les concordances entre les différentes méthodes de reconstruction et la méthode de référence qu'est la méthode directe IDIF, seront évaluées à l'aide de représentations de Bland et Altman et par estimation de coefficients de corrélation intraclasse.

C- La concordance entre les paramètres quantitatifs issus de l'acquisition TEP-FDG LAFOV avec ceux issus d'une reconstruction post-acquisition dégradée TEP SAFOV « like » sera évaluée à l'aide de représentations de Bland et Altman et par estimation de coefficients de corrélation intraclasse.

D- Les facteurs associés à la survie globale seront recherchés à l'aide d'un modèle de Cox, ceux associés à la réponse objective à l'aide d'un modèle logistique.

E- La tolérance et la qualité de vie feront l'objet d'une analyse descriptive en termes de fréquences, moyennes, médianes, quartiles.

Une première analyse statistique sera réalisée après l'inclusion des 40 premiers patients. Etant donné le caractère exploratoire de l'étude, aucun ajustement pour multiplicité des tests statistiques ne sera réalisé pour les analyses.

7.4 Méthode de prise en compte des données manquantes, inutilisées ou non valides

Les analyses seront effectuées sur les données existantes.

7.5 Choix des personnes prise à inclure dans l'analyse

Tous les patients ayant réalisé un TEP LAFOV et reçu au moins une cure de chimio-immunothérapie.

8. TRAITEMENT DES DONNEES ET CONSERVATION DES DOCUMENTS ET DES DONNEES RELATIVES A LA RECHERCHE

8.1. Cahier d'observation

Un tableur Excel anonymisé sera constitué afin de recueillir les données.

8.2. Identification des données recueillies dans le cahier d'observation

- Données démographiques : Nom (première lettre), Prénom (première lettre), N° d'inclusion, âge au diagnostic (année) et sexe.

- Données cliniques baseline :
  - Antécédents : broncho-pneumopathie chronique obstructive, maladie veineuse thrombo-embolique
  - Performans Status, statut tabagique : non-fumeur, fumeur, ex-fumeur sevré, exposition professionnelle à l'amiante, perte de poids

- Données biologiques baseline: polynucléaires neutrophiles, lymphocytes, éosinophiles, albumine, LDH,

- Données histo-pathologiques :
  - Sous-type histologique, statut PD-L1, biologie moléculaire (KRAS, BRAF, HER2, MET, NTRK, NRG1, KEAP1, NFE2L2, STK11, TP53, SMARCA4 + autres marqueurs d'intérêt).
  - Stade du cancer au diagnostic (TNM) : localement avancé non résécable, non irradiable ou métastatique
  - Sites métastatiques

- Données thérapeutiques :
  - Traitement local préalable ou en concomitant : chirurgie, radiothérapie avec fractionnement et dose.
  - Traitement systémique reçu :
    - Type : chimiothérapie par doublet de platine + anti-PD-1
    - Date de 1ère cure
    - Date de dernière cure
    - Date et cause d'arrêt de traitement
  - Effets indésirables du traitement systémique et grade selon la classification CTCAE v5.0.
  - Traitements complémentaires : G-CSF, corticothérapie (posologie).
  - Date de progression tumorale
  - Traitements systémiques ultérieurs à la progression

- Données d'imagerie :
  - Date d'imagerie baseline par TEP LAFOV
  - Date d'imagerie de 1ère réponse systémique
  - Date d'imagerie de meilleure réponse systémique et type (réponse complète, réponse partielle, stabilité, progression)
  - Date d'imagerie de progression

- Paramètres d'imagerie TEP-TDM :
  - Radiomique :
    - Standard (1er ordre) :
      - SUV max = valeur maximale du SUV sur la tumeur ;
      - SUV peak = valeur moyenne la plus élevée possible d'un VOI sphérique de 1 cm3 situé dans la tumeur ;

- MTV (metabolic tumor volume) = volume total en mL incluant la tumeur primitive ou les métastases, mesuré à l'aide de différentes méthodes du seuil de 40 % SUVmax ;
- TLG (total lesion glycolysis) en grammes (g) défini comme MTV x SUVmean (automatiquement calculé comme le SUV moyen dans chaque VOI) ;

- Textural features (2e et 3e ordres) :
  - Entropie, homogénéité, HGZE, LGZE, LRE et SRE.

- Dynamique :

A partir d'un modèle de régression linéaire du graphique Patlak :

- Ki (net influx rate) = coefficient d'absorbtion tumorale en ml/min/100ml
- DV (distribution volume) = volume de distribution en %

- Suivi :
  - Date des dernières nouvelles
  - Si décédé, date du décès.

8.3 Confidentialité

Les documents source étant définis comme tout document ou objet original permettant de prouver l'existence ou l'exactitude d'une donnée ou d'un fait enregistrés au cours de l'étude seront conservés par l'investigateur ou par l'hôpital s'il s'agit d'un dossier médical hospitalier.

Les personnes ayant un accès direct prendront toutes les précautions nécessaires en vue d'assurer la confidentialité des informations relatives aux personnes qui s'y prêtent et notamment en ce qui concerne leur identité ainsi qu'aux résultats obtenus.
Ces personnes, au même titre que les investigateurs eux-mêmes, sont soumises au secret professionnel (selon les conditions définies par les articles 226-13 et 226-14 du code pénal).
Pendant la recherche ou à son issue, les données recueillies sur les personnes qui s'y prêtent et transmises par les intervenants seront rendues codées.
Elles ne doivent en aucun cas faire apparaître en clair les noms des personnes concernées ni leur adresse.

Seule la première lettre du nom et du prénom du sujet seront enregistrées, accompagnées d'un numéro codé propre à l'étude indiquant l'ordre d'inclusion des sujets.

Une table de correspondance papier sera constituée dans chaque centre comprenant le numéro du sujet, la première initiale du nom et du prénom, l'identité complète du sujet, sa date de naissance complète et sa date d'inclusion. Elle sera conservée dans le classeur investigateur jusqu'à 5 ans après la durée de l'étude.

8.4. Déclaration CNIL

Cette étude entre dans le cadre de la « Méthodologie de Référence » (MR-003) en application des dispositions de l'article 54 alinéa 5 de la loi n°78-17 du 6 janvier 1978 modifiée relative à l'informatique, aux fichiers et aux libertés. Ce changement a été homologué par décision °2018-154 du 03 mai 2018. Le CHU de Brest, promoteur de l'étude, a signé un engagement de conformité à cette « Méthodologie de Référence ».

8.5. Traitement des données

La collecte des données cliniques reposera sur la mise en place d'une base de données clinique informatique à partir d'un CRF électronique.
Les incohérences détectées donneront lieu à la production de demandes pour clarification et corrections éventuelles.

La saisie des données sera effectuée par le Dr Margaux Geier et le Dr Karim Amrane sur un CRF électronique accessible par un identifiant et mot de passe.
L'accès au fichier sera verrouillé par un mot de passe pour des raisons supplémentaires de sécurité.
Le Pr Ronan Abgral, co-investigateur, aura également accès à l'intégralité des données.

Circuit des données :

Le Dr Margaux Geier se déplacera en personne dans les structures afin de centraliser l'ensemble des données.

8.6. Archivage des données

Les données du fichier informatique pourront être conservées par le professionnel intervenant dans la recherche sur le réseau de partage de l'établissement jusqu'à deux ans après la dernière publication des résultats de la recherche ou en cas d'absence de publication des résultats jusqu'à signature du résumé final de la recherche.
Par ailleurs, les données du fichier informatique ainsi que tous les documents de l'étude devront faire l'objet d'un archivage par le professionnel intervenant dans la recherche jusqu'à 5 ans après la fin de l'étude.

L'archivage consiste en la conservation d'une copie des données sur support informatique ainsi que tous les documents de l'étude dans une armoire à clef.

9. ASPECT ETHIQUES ET LEGAUX

9.1. Obligations légales.

9.1.1. Rôle du promoteur

Le CHU de Brest est le promoteur de cette recherche conformément au 3ème alinéa de l'article L. 1121-1 du Code de la Santé Publique.
Le promoteur enregistre cette recherche à l'ANSM et soumet le dossier à l'avis du Comité de Protection des Personnes tiré au sort.
Il adressera une copie de l'avis du CPP et du résumé à l'autorité compétente.
Dès la première inclusion, le promoteur informera sans délai le CPP de la date effective de démarrage de l'étude (Date effective de démarrage = date de signature du consentement par la première personne qui se prête à la recherche).
La date de fin d'étude sera transmise par le promoteur au CPP dans un délai de 90 jours. La date de fin de la recherche correspond au terme de la participation de la dernière personne qui se prête à la recherche, ou le cas échéant, au terme défini dans le protocole.

9.1.2. Soumission au CPP

L'avis du CPP est notifié dans la note d'information donnée aux personnes concernées.

9.1.3. Information des centres associés

Le promoteur est chargé d'obtenir l'accord de l'ensemble des parties impliquées dans la recherche.

9.2. Modification de la recherche.

Toute modification substantielle au protocole de l'étude devra être notifiée au Comité de Protection des Personnes par le promoteur afin de vérifier que les modifications proposées n'altèrent à aucun moment les garanties apportées aux personnes qui se prêtent à la recherche.

9.3. Résumé final de l'étude

Le résumé final de la recherche sera écrit par l'investigateur principal/coordonnateur en collaboration avec le statisticien. Après la fin effective de la recherche, il sera remis au promoteur dans les meilleurs délais.

10. PUBLICATION ET PROPRIETE DES DONNEES

Le CHU de Brest est propriétaire des données et aucune utilisation ou transmission à un tiers ne peut être effectuée sans accord préalable. Les termes de « CHU de Brest » doit apparaître dans l'adresse des auteurs.
Les communications et rapports scientifiques correspondant à cette étude seront réalisés sous la responsabilité de l'investigateur principal coordonnateur de l'étude. Les coauteurs du rapport et des publications seront les investigateurs et les cliniciens impliqués, au prorata de leur contribution à l'étude, ainsi que le biostatisticien et les chercheurs associés.
Les règles de publications suivront les recommandations internationales (N Engl J Med, 1997; 336 :309-315).
L'étude sera enregistrée sur un site web en libre accès (Clinical trial) avant l'inclusion du 1er patient dans cette étude.

11. BIBLIOGRAPHIE

1. Siegel RL, Miller KD, Jemal A. Cancer statistics, 2018. CA Cancer J Clin. 2018 Jan;68(1):7-30. doi: 10.3322/caac.21442.

2. Sung H, Ferlay J, Siegel RL, et al. Global Cancer Statistics 2020: GLOBOCAN Estimates of Incidence and Mortality Worldwide for 36 Cancers in 185 Countries. CA Cancer J Clin. 2021 May;71(3):209-249. doi: 10.3322/caac.21660.

3. Debieuvre D, Molinier O, Falchero L et al; Study Group KBP-2020-CPHG; KBP-2020-CPHG. Lung cancer trends and tumor characteristic changes over 20 years (2000-2020): Results of three French consecutive nationwide prospective cohorts' studies. Lancet Reg Health Eur. 2022 Aug 29;22:100492. doi: 10.1016/j.lanepe.2022.100492.

4. Brahmer J, Reckamp KL, Baas P, et al. Nivolumab versus Docetaxel in Advanced Squamous-Cell Non-Small-Cell Lung Cancer. N Engl J Med. 2015 Jul 9;373(2):123-35. doi: 10.1056/NEJMoa1504627.

5. Borghaei H, Paz-Ares L, Horn L, et al. Nivolumab versus Docetaxel in Advanced Nonsquamous Non-Small-Cell Lung Cancer. N Engl J Med. 2015 Oct 22;373(17):1627-39. doi: 10.1056/NEJMoa1507643.

6. Herbst RS, Baas P, Kim DW, et al. Pembrolizumab versus docetaxel for previously treated, PD-L1-positive, advanced non-small-cell lung cancer (KEYNOTE-010): a randomised controlled trial. Lancet. 2016 Apr 9;387(10027):1540-1550. doi: 10.1016/S0140-6736(15)01281-7.

7. Gandhi L, Rodríguez-Abreu D, Gadgeel S, et al; KEYNOTE-189 Investigators. Pembrolizumab plus Chemotherapy in Metastatic Non-Small-Cell Lung Cancer. N Engl J Med. 2018 May 31;378(22):2078-2092. doi: 10.1056/NEJMoa1801005.

8. Paz-Ares L, Luft A, Vicente D, et al; KEYNOTE-407 Investigators. Pembrolizumab plus Chemotherapy for Squamous Non-Small-Cell Lung Cancer. N Engl J Med. 2018 Nov 22;379(21):2040-2051. doi: 10.1056/NEJMoa1810865.

9. Garassino MC, Gadgeel S, Speranza G, et al. Pembrolizumab Plus Pemetrexed and Platinum in Nonsquamous Non-Small-Cell Lung Cancer: 5-Year Outcomes From the Phase 3 KEYNOTE-189 Study. J Clin Oncol. 2023 Apr 10;41(11):1992-1998. doi: 10.1200/JCO.22.01989.

10. Novello S, Kowalski DM, Luft A, et al. Pembrolizumab Plus Chemotherapy in Squamous Non-Small-Cell Lung Cancer: 5-Year Update of the Phase III KEYNOTE-407 Study. J Clin Oncol. 2023 Apr 10;41(11):1999-2006. doi: 10.1200/JCO.22.01990.

11. Dagogo-Jack I, Shaw AT. Tumour heterogeneity and resistance to cancer therapies. Nat Rev Clin Oncol. 2018 Feb;15(2):81-94. doi: 10.1038/nrclinonc.2017.166.

12. Borghaei H, Langer CJ, Paz-Ares L, et al. Pembrolizumab plus chemotherapy versus chemotherapy alone in patients with advanced non-small cell lung cancer without tumor PD-L1 expression: A pooled analysis of 3 randomized controlled trials. Cancer. 2020 Nov 15;126(22):4867-4877. doi: 10.1002/cncr.33142.

13. Salaün PY, Abgral R, Malard O, et al. Good clinical practice recommendations for the use of PET/CT in oncology. Eur J Nucl Med Mol Imaging. 2020 Jan;47(1):28-50. doi: 10.1007/s00259-019-04553-8.

14. Abgral R, Bourhis D, Salaun PY. Clinical perspectives for the use of total body PET/CT. Eur J Nucl Med Mol Imaging. 2021 Jun;48(6):1712-1718. doi: 10.1007/s00259-021-05293-4.

15. Hatt M, Tixier F, Pierce L, et al. Characterization of PET/CT images using texture analysis: the past, the present… any future? Eur J Nucl Med Mol Imaging. 2017 Jan;44(1):151-165. doi: 10.1007/s00259-016-3427-0.

16. Manafi-Farid R, Karamzade-Ziarati N, Vali R, et al. 2-[^{18}F]FDG PET/CT radiomics in lung cancer: An overview of the technical aspect and its emerging role in management of the disease. Methods. 2021 Apr;188:84-97. doi: 10.1016/j.ymeth.2020.05.023.

17. Reuzé S, Schernberg A, Orlhac F, et al. Radiomics in Nuclear Medicine Applied to Radiation Therapy: Methods, Pitfalls, and Challenges. Int J Radiat Oncol Biol Phys. 2018 Nov 15;102(4):1117-1142. doi: 10.1016/j.ijrobp.2018.05.022.

18. Zaidi H, Karakatsanis N. Towards enhanced PET quantification in clinical oncology. Br J Radiol. 2018 Jan;91(1081):20170508. doi: 10.1259/bjr.20170508.

19. Karakatsanis NA, Lodge MA, Tahari AK, Zhou Y, Wahl RL, Rahmim A. Dynamic whole-body PET parametric imaging: I. Concept, acquisition protocol optimization and clinical application. Phys Med Biol. 2013 Oct 21;58(20):7391-418. doi: 10.1088/0031-9155/58/20/7391.

20. Pavoine M, Thuillier P, Karakatsanis N et al. Clinical application of a population-based input function (PBIF) for a shortened dynamic whole-body FDG-PET/CT protocol in patients with metastatic melanoma treated by immunotherapy. EJNMMI Phys. 2023 Dec 8;10(1):79. doi: 10.1186/s40658-023-00601-3.

21. Thuillier P, Bourhis D, Metges JP, et al. Prospective study of dynamic whole-body 68Ga-DOTATOC-PET/CT acquisition in patients with well-differentiated neuroendocrine tumors. Sci Rep. 2021 Mar 1;11(1):4727. doi: 10.1038/s41598-021-83965-9.

22. Sari, H., Teimoorisichani, M., Mingels, C. et al. Quantitative evaluation of a deep learning-based framework to generate whole-body attenuation maps using LSO background radiation in long axial FOV PET scanners. Eur J Nucl Med Mol Imaging 49, 4490–4502 (2022). https://doi.org/10.1007/s00259-022-05909-3

23. Wang D, Qiu B, Liu Q, et al. Patlak-Ki derived from ultra-high sensitivity dynamic total body [18F]FDG PET/CT correlates with the response to induction immuno-chemotherapy in locally advanced non-small cell lung cancer patients. Eur J Nucl Med Mol Imaging. 2023 Sep;50(11):3400-3413. doi: 10.1007/s00259-023-06298-x.

24. Socinski MA, Nishio M, Jotte RM, et al. IMpower150 Final Overall Survival Analyses for Atezolizumab Plus Bevacizumab and Chemotherapy in First-Line Metastatic Nonsquamous NSCLC. J Thorac Oncol. 2021 Nov;16(11):1909-1924. doi: 10.1016/j.jtho.2021.07.009. Epub 2021 Jul 24. PMID: 34311108.

25. Eisenhauer EA, Therasse P, Bogaerts J, et al. New response evaluation criteria in solid tumours: revised RECIST guideline (version 1.1). Eur J Cancer. 2009 Jan;45(2):228–47.

26. Wahl RL, Jacene H, Kasamon Y et al. From RECIST to PERCIST: Evolving Considerations for PET response criteria in solid tumors. J Nucl Med. 2009 May;50 Suppl 1(Suppl 1):122S-50S. doi: 10.2967/jnumed.108.057307.

27. 16. Common Terminology Criteria for Adverse Events (CTCAE). Available from : https://ctep.cancer.gov/protocolDevelopment/electronic_applications/docs/CTCAE_v5_QuickReference_8.5x11.pdf
