# Supplementary material for: PRognostic and predictive potential Of multiparametric dynamic whole-body 18F-FDG PET Imaging using a Long axial field-of-view (LAFOV) system for FIRST-line chemo-immunotherapy efficacy in advanced non-small cell lung cancer: PROFIL-1 study protocol
Source: PLoS One. 2026 Apr 3;21(4):e0345990. doi: 10.1371/journal.pone.0345990 (PMC13048445; doi:10.1371/journal.pone.0345990)
Supplement: S3 File — Completed SPIRIT 2013 checklist for the PROFIL-1 study protocol. (PDF) [file pone.0345990.s003.pdf]

SPIRIT 2025 checklist of items to address in a randomized trial protocol*

Section / Topic	No	SPIRIT 2025 checklist item description	Reported on page no.
**Administrative information**			
Title and structured summary	1a	Title stating the trial design, population, and interventions, with identification as a protocol	1; lines 1-4
	1b	Structured summary of trial design and methods, including items from the World Health Organization Trial Registration Data Set	2; lines 34-60
Protocol version	2	Version date and identifier	2; lines 31-32
Roles and responsibilities	3a	Names, affiliations, and roles of protocol contributors	1; lines 6-29 24-25; lines 693-704
	3b	Name and contact information for the trial sponsor	N/A
	3c	Role of trial sponsor and funders in design, conduct, analysis, and reporting of trial; including any authority over these activities	N/A
	3d	Composition, roles, and responsibilities of the coordinating site, steering committee, endpoint adjudication committee, data management team, and other individuals or groups overseeing the trial, if applicable *> The study is coordinated by the Department of Medical Oncology, University Hospital of Brest (principal investigator: Dr Margaux Geier). No steering committee, endpoint adjudication committee, or data monitoring committee has been established, as the study is non-interventional.*	7; lines 180-184
**Open science**			
Trial registration	4	Name of trial registry, identifying number (with URL), and date of registration. If not yet registered, name of intended registry	13; lines 363-370
Protocol and statistical analysis plan	5	Where the trial protocol and statistical analysis plan can be accessed	12-13; lines 338-361
Data sharing	6	Where and how the individual de-identified participant data (including data dictionary), statistical code, and any other materials will be accessible	N/A
Funding and conflicts of interest	7a	Sources of funding and other support (e.g., supply of drugs)	24, lines 688-690
	7b	Financial and other conflicts of interest for principal investigators and steering committee members	24; lines 685-686
Dissemination policy	8	Plans to communicate trial results to participants, healthcare professionals, the public, and other relevant groups (e.g., reporting in trial registry, plain language summary, publication)	13: lines 369-370
**Introduction**			
Background and rationale	9a	Scientific background and rationale, including summary of relevant studies (published and unpublished) examining benefits and harms for each intervention	4-5; lines 87-147
	9b	Explanation for choice of comparator	N/A
Objectives	10	Specific objectives related to benefits and harms	5; lines 141-147

**Methods: Patient and public involvement, trial design**			
Patient and public involvement	11	Details of, or plans for, patient or public involvement in the design, conduct, and reporting of the trial	8-9; lines 206-250
Trial design	12	Description of trial design including type of trial (e.g., parallel group, crossover), allocation ratio, and framework (e.g., superiority, equivalence, non-inferiority, exploratory)	6; lines 176-177
**Methods: Participants, interventions, and outcomes**			
Trial setting	13	Settings (e.g., community, hospital) and locations (e.g., countries, sites) where the trial will be conducted	7; lines 180-184
Eligibility criteria	14a	Eligibility criteria for participants	7; lines 193-196
	14b	If applicable, eligibility criteria for sites and for individuals who will deliver the interventions (e.g., surgeons, physiotherapists)	N/A
Intervention and comparator	15a	Intervention and comparator with sufficient details to allow replication including how, when, and by whom they will be administered. If relevant, where additional materials describing the intervention and comparator (e.g., intervention manual) can be accessed	N/A
	15b	Criteria for discontinuing or modifying allocated intervention/comparator for a trial participant (e.g., drug dose change in response to harms, participant request, or improving/worsening disease)	N/A
	15c	Strategies to improve adherence to intervention/comparator protocols, if applicable, and any procedures for monitoring adherence (e.g., drug tablet return, sessions attended)	N/A
	15d	Concomitant care that is permitted or prohibited during the trial	N/A
Outcomes	16	Primary and secondary outcomes, including the specific measurement variable (e.g., systolic blood pressure), analysis metric (e.g., change from baseline, final value, time to event), method of aggregation (e.g., median, proportion), and time point for each outcome	9-10; lines 257-284
Harms	17	How harms are defined and will be assessed (e.g., systematically, non-systematically)	N/A
Participant timeline	18	Time schedule of enrollment, interventions (including any run-ins and washouts), assessments, and visits for participants. A schematic diagram is highly recommended (see Figure)	8 (figure 1A/1B) lines 206-209
Sample size	19	How sample size was determined, including all assumptions supporting the sample size calculation	7; lines 186-191
Recruitment	20	Strategies for achieving adequate participant enrollment to reach target sample size	7; lines 183-184
**Methods: Assignment of interventions**			
Randomization:			N/A
Sequence generation	21a	Who will generate the random allocation sequence and the method used	N/A
	21b	Type of randomization (simple or restricted) and details of any factors for stratification. To reduce predictability of a random sequence, other details of any planned restriction (e.g., blocking) should be	N/A

		provided in a separate document that is unavailable to those who enroll participants or assign interventions	
Allocation concealment mechanism	22	Mechanism used to implement the random allocation sequence (e.g., central computer/telephone; sequentially numbered, opaque, sealed containers), describing any steps to conceal the sequence until interventions are assigned	N/A
Implementation	23	Whether the personnel who will enroll and those who will assign participants to the interventions will have access to the random allocation sequence	N/A
Blinding	24a	Who will be blinded after assignment to interventions (e.g., participants, care providers, outcome assessors, data analysts)	N/A
	24b	If blinded, how blinding will be achieved and description of the similarity of interventions	N/A
	24c	If blinded, circumstances under which unblinding is permissible, and procedure for revealing a participant's allocated intervention during the trial	N/A
**Methods: Data collection, management, and analysis**			
Data collection methods	25a	Plans for assessment and collection of trial data, including any related processes to promote data quality (e.g., duplicate measurements, training of assessors) and a description of trial instruments (e.g., questionnaires, laboratory tests) along with their reliability and validity, if known. Reference to where data collection forms can be accessed, if not in the protocol	11-12; lines 286-336
	25b	Plans to promote participant retention and complete follow-up, including list of any outcome data to be collected for participants who discontinue or deviate from intervention protocols	8; lines 206-209
Data management	26	Plans for data entry, coding, security, and storage, including any related processes to promote data quality (e.g., double data entry; range checks for data values). Reference to where details of data management procedures can be accessed, if not in the protocol	11; lines 331-336
Statistical methods	27a	Statistical methods used to compare groups for primary and secondary outcomes, including harms	N/A
	27b	Definition of who will be included in each analysis (e.g., all randomized participants), and in which group	N/A
	27c	How missing data will be handled in the analysis	N/A
	27d	Methods for any additional analyses (e.g., subgroup and sensitivity analyses)	12-13; lines 338-361
**Methods: Monitoring**			
Data monitoring committee	28a	Composition of data monitoring committee (DMC); summary of its role and reporting structure; statement of whether it is independent from the sponsor and funder; conflicts of interest and reference to where further details about its charter can be found, if not in the protocol. Alternatively, an explanation of why a DMC is not needed	N/A
	28b	Explanation of any interim analyses and stopping guidelines, including who will have access to these interim results and make the final decision to terminate the trial	N/A
Trial monitoring	29	Frequency and procedures for monitoring trial conduct. If there is no monitoring, give explanation	7; lines 175-177<br>As this is a non-interventional observational study, no formal on-site monitoring visits are planned. Data quality and protocol compliance will be ensured through centralized data control by the sponsor (University

			Hospital of Brest) and regular internal reviews of the electronic case report forms. Any protocol deviations will be documented and reviewed by the coordinating investigator.
**Ethics**			
Research ethics approval	30	Plans for seeking research ethics committee/institutional review board approval	13-14; lines 363-374
Protocol amendments	31	Plans for communicating important protocol modifications to relevant parties	13-14; lines 363-374
Consent or assent	32a	Who will obtain informed consent or assent from potential trial participants or authorized proxies, and how	8; lines 197-204
	32b	Additional consent provisions for collection and use of participant data and biological specimens in ancillary studies, if applicable	N/A
Confidentiality	33	How personal information about potential and enrolled participants will be collected, shared, and maintained in order to protect confidentiality before, during, and after the trial	12; lines 331-336
Ancillary and post-trial care	34	Provisions, if any, for ancillary and post-trial care, and for compensation to those who suffer harm from trial participation	N/A

*We strongly recommend reading this checklist in conjunction with the SPIRIT 2025 Explanation and Elaboration and the SPIRIT 2025 Expanded Checklist for important clarifications on all the items. We also recommend reading relevant SPIRIT extensions. See www.consort-spirit.org

Citation: Chan A-W, Boutron I, Hopewell S, Moher D, Schulz KF, et al. SPIRIT 2025 statement: updated guideline for protocols of randomised trials. BMJ 2025;389:e081477. https://dx.doi.org/10.1136/bmj-2024-081477

© 2025 Chan A-W et al. This is an Open Access article distributed under the terms of the Creative Commons Attribution License (https://creativecommons.org/licenses/by/4.0/), which permits unrestricted use, distribution, and reproduction in any medium, provided the original work is properly cited.
